# Immunization with recombinant truncated *Neisseria meningitidis*-Macrophage Infectivity Potentiator (rT-Nm-MIP) protein induces murine antibodies that are cross-reactive and bactericidal for *Neisseria gonorrhoeae*

**DOI:** 10.1016/j.vaccine.2018.05.069

**Published:** 2018-06-22

**Authors:** María Victoria Humbert, Myron Christodoulides

**Affiliations:** Neisseria Research Group, Molecular Microbiology, Academic Unit of Clinical and Experimental Sciences, Sir Henry Wellcome Laboratories, University of Southampton, Faculty of Medicine, Southampton SO166YD, United Kingdom

**Keywords:** Macrophage Infectivity Potentiator, *Neisseria gonorrhoeae*, *Neisseria meningitidis*, Bactericidal antibody, Human complement, Liposomes

## Abstract

*Neisseria meningitidis* (Nm) and *N. gonorrhoeae* (Ng) express a Macrophage Infectivity Potentiator (MIP, NMB1567/NEIS1487) protein in their outer membrane (OM). In this study, we prepared independent batches of liposomes (n = 3) and liposomes + MonoPhosphoryl Lipid A (MPLA) (n = 3) containing recombinant truncated Nm-MIP protein encoded by Allele 2 (rT-Nm-MIP, amino acids 22–142), and used these to immunize mice. We tested the hypothesis that independent vaccine batches showed similar antigenicity, and that antisera could recognise both meningococcal and gonococcal MIP and induce cross-species bactericidal activity.

The different batches of M2 rT-Nm-MIP-liposomes ± MPLA showed no significant (P > 0.05) batch-to-batch variation in antigenicity. Anti-rT-Nm-MIP sera reacted equally and specifically with Nm-MIP and Ng-MIP in OM and on live bacterial cell surfaces. Specificity was shown by no antiserum reactivity with *Δmip* bacteria. Using human complement/serum bactericidal assays, anti-M2 rT-Nm-MIP sera killed homologous meningococcal serogroup B (MenB) strains (median titres of 32–64 for anti-rT-Nm-MIP-liposome sera; 128–256 for anti-rT-Nm-MIP-liposome + MPLA sera) and heterologous M1 protein-expressing MenB strains (titres of 64 for anti rT-Nm-MIP-liposome sera; 128–256 for anti-rT-Nm-MIP-liposome + MPLA sera). Low-level killing (P < 0.05) was observed for a MenB isolate expressing M7 protein (titres 4–8), but MenB strains expressing M6 protein were not killed (titre < 4–8). Killing (P < 0.05) was observed against MenC and MenW bacteria expressing homologous M2 protein (titres of 8–16) but not against MenA or MenY bacteria (titres < 4–8).

Antisera to M2 rT-Nm-MIP showed significant (P < 0.05) cross-bactericidal activity against gonococcal strain P9-17 (expressing M35 Ng-MIP, titres of 64–512) and strain 12CFX_T_003 (expressing M10 Ng-MIP, titres 8–16) but not against FA1090 (expressing M8 Ng-MIP).

As an alternative to producing recombinant protein, we engineered successfully the Nm-OM to express M2 Truncated–Nm-MIP, but lipooligosaccharide-extraction with Na-DOC was contra-indicated. Our data suggest that a multi-component vaccine containing a select number of Nm- and Ng-MIP type proteins would be required to provide broad coverage of both pathogens.

## Highlights

• Antigenicity of rT-*N. meningitidis-*MIP vaccine batches is reproducible in mice.
• Antibodies to rT-Nm*-*MIP cross-react with surface Ng-MIP and adhere to gonococci.
• Antisera to rT-Nm-MIP are cross-bactericidal for gonococci.
• Meningococcal OM can be engineered to express T-Nm-MIP.

## 1 Introduction

*Neisseria meningitidis* (Nm, Men, meningococcus) is a major causative organism of meningitis and sepsis contributing significantly to mortality and morbidity worldwide [1], and *Neisseria gonorrhoeae* (Ng, gonococcus) causes the sexually transmitted disease gonorrhoea [2]. Capsular polysaccharide-conjugate vaccines to prevent infections by MenA, MenC, MenW and MenY meningococci are widely available, routinely used and effective [3], [4], and two new vaccines Bexsero and Trumenba, have been licensed for MenB infection [5], [6]. Bexsero has shown a vaccine efficacy of 83% against all MenB cases in vaccine-eligible infants in the UK since 2015, equivalent to ∼94% efficacy against the 88% predicted vaccine-preventable MenB strains [7]. A 50% reduction in MenB cases was observed in the vaccine eligible age-group, which seems to have persisted [8]. By contrast, there are no gonorrhoea vaccines and infection control has relied on antibiotics, but this is being severely compromised by the emergence of antibiotic-resistant gonococci worldwide [9]. Thus, new vaccine technologies have led to renewed interest in developing prophylactic gonococcal vaccines [10], [11].

The impact of new MenB vaccines on the levels of protection, the epidemiology of circulating meningococcal strains, the potential for selection of new antigenic variants and variation in protein expression, needs to be monitored. Next-generation MenB vaccines may see the incorporation of additional antigens capable of inducing serum bactericidal antibodies, the accepted correlate of protection. A possible candidate for inclusion in new vaccines is the Macrophage Infectivity Potentiator protein (MIP, NMB1567/NEIS1487), which is a member of the FK506-binding protein (FKBP)-type peptidyl prolyl cis/trans isomerase (PPIase) family of proteins [12], [13]. Expression of the *nm-mip* gene was important for meningococcal survival in the blood [13], [14] and inhibition of Nm-MIP prevented meningococci from adhering, invading and/or surviving in epithelial cells [15]. Nm-MIP is highly conserved and expressed by all meningococcal strains reported to date, is outer membrane (OM)-located, surface exposed and capable of inducing cross-protective bactericidal antibodies [13], [16], [17]. *N. gonorrhoeae* also produces a FKBP-type PPIase and expression of the surface-exposed 30 kDa Ng-MIP lipoprotein appeared to be important for bacterial persistence within macrophages and protected gonococci from the bactericidal activity of immune effector cells [18], [19]. Sera from patients with urethritis or disseminated gonococcal infections recognized purified Ng-MIP, suggesting that this antigen is expressed during infection *in vivo* and is immunogenic [13], [18], [19]. Ng-MIP is also highly conserved across all reported strains of *N. gonorrhoeae*, although the vaccine potential has not been reported.

Recently, we reported that Nm-MIP and human FKBP2 PPIase protein shared ∼48% similarity of amino acids (AA) located in region AA166-252. The C-terminal globular domain of Nm-MIP covers AA143-272 and contains the PPIase FKBP-type domain [16]. Molecular mimicry between Nm-MIP with hFKBP2 protein was obviated by generating a recombinant truncated protein (rT-Nm-MIP, AA22-143), which induced murine bactericidal antibodies against meningococci that did not recognise human FKBP protein [16]. A baby rabbit complement Serum Bactericidal Assay (BRC-SBA) demonstrated that antibodies to rT-Nm-MIP Type I protein were bactericidal for MenB bacteria expressing different MIP (Type I, II and III) proteins and for MenA, MenC, MenW and MenY bacteria expressing the same MIP protein. Antisera to rT-Nm-MIP appeared also to show bactericidal activity against a gonococcal strain P9-17, although the BRC-SBA showed high levels of background killing by sham-immunized sera. For examining bactericidal activity of antisera raised to recombinant proteins, it is preferable that MenB and gonococcal SBAs use a human complement/human serum (HC/HS) source rather than BRC, which tends to inflate bactericidal titres due to the presence of IgM antibodies. It has been reported also that the HC-SBA is conservative with a high rate of false-negatives, which makes the assay less sensitive, but more specific, that the BRC-SBA [20], [21], [22].

In the current study, we extended our studies of the vaccine potential of rT-Nm-MIP by (i) examining the antigenicity of independent batches of experimental vaccines against meningococci and gonococci, (ii) using HC/HS-SBA assays to quantify bactericidal activity against meningococci and gonococci expressing different MIP Type proteins and (iii) attempting to engineer the MenB OM to express truncated MIP.

## 2 Materials and methods

### 2.1 Bacteria, growth conditions and preparation of outer membranes (OM)

Bacteria used in this study are listed in Table 1. Wild type and mutant strains were grown on supplemented GC agar plates, incubated at 37 °C, 5% (v/v) CO_2_ [23]. For human complement-mediated serum bactericidal assays (HC-SBA), *N. gonorrhoeae* strains MS11 and 12CFX_T_003 were grown on supplemented GC agar plates with the addition of Cytidine-5′-MonoPhospho-N-AcetylNeuraminic Acid to impart resistance to human serum (HS) [24]. *Escherichia coli* DH5α (cloning) and BL21(DE3) pLysS strains (protein expression) were grown at 37 °C on Luria-Bertani (LB) agar, LB or SOB broths.

**Table 1:** *Neisseria* organisms used in this study. NIPH, Norwegian Institute of Public Health, Norway. ATCC, American Type Culture Collection. PHE, Public Health England. CDCP/FDA – Centre for Disease Control and Prevention/Food and Drug Administration Antibiotic/Antimicrobial Resistance Isolate Bank (https://www.cdc.gov/drugresistance/resistance-bank/currently-available.html).

Organism	Strain	Serogroup	Provenance
*Neisseria meningitidis*	Z1534	A	NIPH, Norway
	Z1092	A	NIPH, Norway
	MC58	B	[16]
	MC168	B	[16]
	MC90	B	[16]
	MC54	B	[16]
	M15 240139	B	PHE, Manchester
	M15 240337	B	PHE, Manchester
	M15 240973	B	PHE, Manchester
	M16 240169	B	PHE, Manchester
	MC173	C	[23]
	M11 240441	W	PHE, Manchester
	M12 240717	Y	PHE, Manchester
	M15 240043	Y	PHE, Manchester
	M16 240363	Y	PHE, Manchester

*Neisseria gonorrhoeae*	P9-17	–	[39]
	FA1090	–	ATCC700825
	MS11	–	ATCC BA1833
	12CFX_T_003	–	CDCP/FDA - AR Isolate Bank

OM of MC58, MC58Δm*ip* and MC58Δm*ip::t-nm-mip*, P9-17 and FA1090 bacteria were prepared as described previously [25], [26]. Treatment with sodium deoxycholate (Na-DOC) was done as described previously [26].

### 2.2 Construction of Neisseria meningitidis nm-mip gene and Neisseria gonorrhoeae ng-mip gene knock-out mutants

Construction of MC58Δm*ip*, FA1090Δm*ip* and P9-17Δm*ip* mutant strains was done as described previously [14], [16] using primers listed in Supplementary Table 1. Transformed colonies were screened by PCR and confirmed by western blotting using cross-reacting rabbit anti-rNm-MIP sera [17].

### 2.3 Complementation of MC58Δnm-mip strain with c-term truncated nm-mip

For chromosomal complementation of MC58Δm*ip* strain, the 3′-end truncated *nm-mip* gene (encoding for AA1-143) under transcriptional regulation of a strong and constitutive *PorA/NadA* hybrid promoter [27] was engineered *in silico* into pMA-T (GeneArt) and subsequently cloned into pGCC5. This construct was used to complement MC58Δm*ip* strain [28]. Positive transformants were identified by PCR screening and western blotting with rabbit anti-rNm-MIP sera [17].

### 2.4 Cloning, expression, and purification of recombinant full and T-Nm-MIP (M2, Type I) proteins

Cloning, expression and purification of full-length rNm-MIP (M2, Type I) protein was described previously [17]. The 3′-truncated *nm-mip* gene sequence, encoding the N-terminal amino acid sequence (AA 22–143) for NMB1567 protein (NEIS1487, http://pubmlst.org/neisseria/, 819 bp), was amplified by PCR using primers listed in Supplementary Table 1, and ligated to pET24b+. Mature M2 (Type I) rT-Nm-MIP protein (13.93 kDa) was purified by Ni-NTA affinity chromatography under native conditions (Supplementary Fig. 1).

### 2.5 Animal immunizations

Groups of five BALB/c mice (H-2^d^ haplotype) were immunized intraperitoneally with purified M2 rT-Nm-MIP protein delivered in liposomes, with or without Monophosphoryl Lipid A (MPLA), or with OM and Na-DOC OM preparations from strains MC58, MC58Δm*ip* and MC58Δm*ip::t-nm-mip* adsorbed to Al(OH)_3_, following the immunization schedule described previously [16]. All sera were stored at -20 °C until required and decomplemented by heating at 56 °C for 30 min before use in HC/HS-SBA.

This study complied with the animal experimentation guidelines of the Home Office (HO), with approval granted under the Animals Scientific Procedures Act, 1986 (HO PPL 3003126). The study was approved by the Animal Welfare and Ethics Review Board at the authors’ institution (no number assigned). Animal health and welfare was assessed daily by qualified animal technicians and no animals suffered significant adverse effects.

### 2.6 Antibody properties

#### 2.6.1 Enzyme-linked immunosorbent assay (ELISA)

Individual murine antisera were reacted with M2 rT-Nm-MIP, MC58, MC58Δm*ip*, MC58Δm*ip::t- nm-mip*, *N. gonorrhoeae* P9-17 and FA1090 OM preparations. Immunological reactivity was detected with anti-mouse immunoglobulin-horseradish peroxidase conjugate, as described previously [16], [17].

#### 2.6.2 SDS-PAGE and western immunoblotting

Immunological reactivity of murine sera to OM preparations was detected with anti-mouse/rabbit immunoglobulin-alkaline phosphatase conjugates, as described previously [16], [17].

#### 2.6.3 Flow cytometry

Binding of antibodies to MC58, MC58Δm*ip*, MC58Δm*ip::t-nm-mip*, P9-17, P9-17Δm*ip*, FA1090 and FA1090Δm*ip* bacteria was examined by flow cytometry, as described previously [16], [17].

#### 2.6.4 Complement-activated killing of Neisseriae

To prepare human complement (HC) source, 2.5 ml of fresh normal HS with the addition of 50 µl of 0.5 M EDTA, pH 8 and 350 µl of 5 M NaCl was incubated first with anti-human IgM resin and then with Protein A/G Plus resin (with rotation) at 4 °C for 1 h each. The final HC eluate was dialyzed twice against filter-sterilized ice-cold TBS, 0.1 mM EDTA and brought back to 2.5 ml in the same buffer. The bactericidal activity of pooled murine anti-rT-Nm-MIP sera was determined as described previously [29], using 25% (v/v) HC for meningococcal strains [22], [30] and 17% (v/v) HS as a source of exogenous complement for gonococcal strains [31].

### 2.7 Statistics

Data were compared using one-sample, two-sample or paired *t*-Tests, as appropriate, with P values < 0.05 considered significant.

## 3 Results

### 3.1 Conservation of NEIS1487 in pathogenic Neisseria species

**Supplementary Fig. 3:**
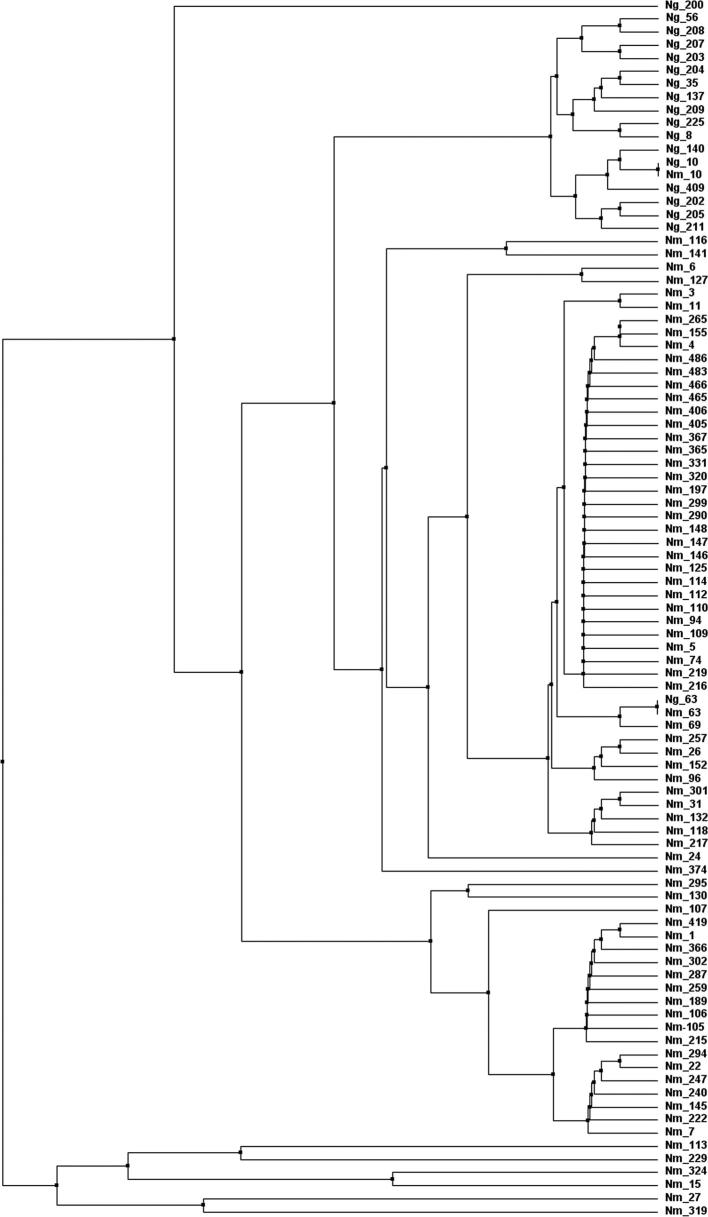
Dendrogram showing the clustering of non-redundant MIP proteins in meningococcal and gonococcal isolates in the pubmlst.org/*Neisseria* database. A dendrogram was assembled using the non-redundant sequences with Jalview 2.9 (www.jalview.org).

The DNA sequences of the *nmb1567*/*neis1487* gene from pathogenic *Neisseria* strains in the pubmlst.org/Neisseria database [32] were translated to amino acid sequences and aligned. The database contains 10,434 meningococcal and 3876 gonococcal isolates (accessed Jan. 2018) with 168 and 33 alleles identified, respectively. Analysis of the translated proteins encoded by the nucleotide sequences of these different alleles identified 75 and 18 non-redundant NEIS1487 amino acid sequences amongst meningococci and gonococci respectively (Supplementary Table 2; Supplementary Figs. 2, 3). The percentage distribution of meningococcal isolates amongst the non-redundant alleles has not changed significantly since a previous analysis [13], with the majority expressing protein encoded by Allele 2 (41%), Allele 1 (32%) and Allele 7 (11%). For gonococci, most isolates expressed protein encoded by Allele 10 (59%) or Allele 35 (27%) and fewer isolates by other alleles *e.g.* Allele 8 (5%) (Supplementary Table 2). Only Allele 10 and 63 were found in both meningococci and gonococci (Supplementary Fig. 3), though only 5 gonococcal isolates expressed Allele 63-encoded protein compared to a total of 115 meningococcal isolates, and conversely only 2 meningococcal isolates (not determined) expressed Allele 10-encoded protein compared to 2274 gonococcal isolates (Supplementary Table 2).

The MIP Type designation scheme was originally based on the isolate frequency for each non-redundant protein, referred to as Types I-IV, with Type I being the most abundant Type protein within all *N. meningitidis* isolates reported [13], [17]. We now re-assign the MIP Type designation by referring to the representative allele encoding for a particular non-redundant MIP protein, thus becoming independent of the isolate frequency ranking for each particular MIP type, which varies as the database grows in number of isolates reported. Thus, in meningococci, M (*i.e.* MIP)2 protein is encoded by Allele 2 (formerly Nm-MIP Type I), M1 protein is encoded by Allele 1 (formerly Nm-MIP Type II), M6 protein is encoded by Allele 6 (formerly Nm-MIP Type III) and M7 protein is encoded by Allele 7 (formerly Nm-MIP Type IV) [13] and in gonococci, M8, M10 and M35 proteins are encoded by Alleles 8, 10 and 35, respectively (Supplementary Table 2).

**Fig. 1:**
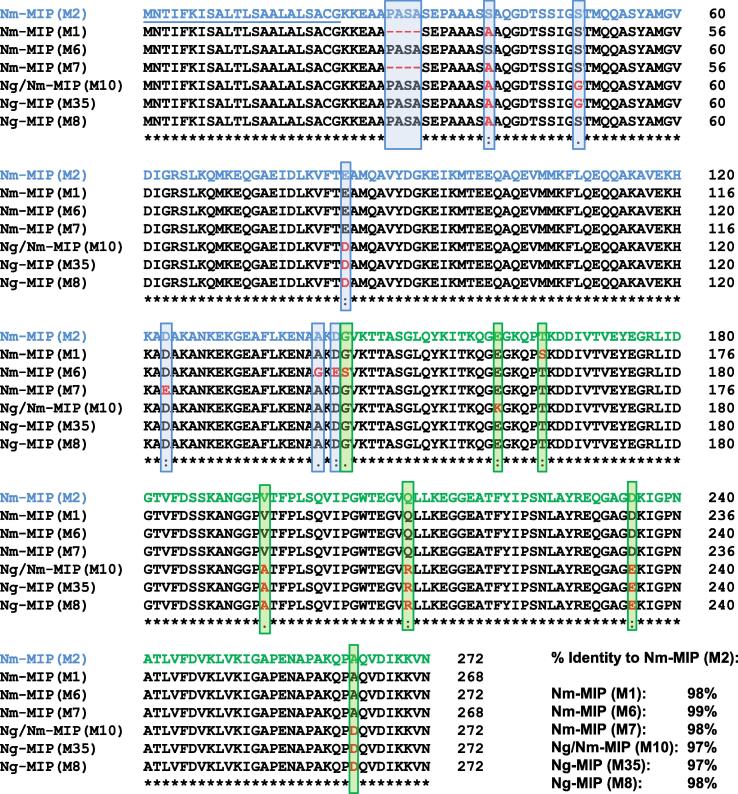
Clustal alignment of representative Nm-MIP and Ng-MIP proteins. Amino acid sequence alignment of Nm-MIP (M1, M2, M6, M7) and Allele 8, 10 and 35-encoded Ng-MIP proteins (M8, M10 and M35). The regions in Nm-MIP M2 (Type I) sequence highlighted in blue and green correspond to residues 1–142 (N-terminal domain) and 143–272 (C-terminal domain), respectively. The leader sequence in Nm-MIP M2 (Type I) (amino acids 1–22) is underlined. Distinct amino acid residues to Nm-MIP M2 (Type I) are highlighted in red and shown within a blue or a green box, depending on whether they belong to the N- or C-terminal domain. The percentage of identity of each protein to M2 (Type I) Nm-MIP is specified. (For interpretation of the references to colour in this figure legend, the reader is referred to the web version of this article.)

Alignment of the amino acid sequences of the most prevalent meningococcal (M1, 2, 6, 7) and gonococcal (M8, 10, 35) allele-encoded proteins showed a high degree of homology between the whole proteins (97–99%, Fig. 1). Within the C-terminal truncated region, homology between the gonococcal sequences and M2 was 98–99% and marginally more variability between the different meningococcal sequences (96–99%) (Supplementary Fig. 4).

### 3.2 Antigenicity of rT-Nm-MIP vaccine batches

**Fig. 2:**
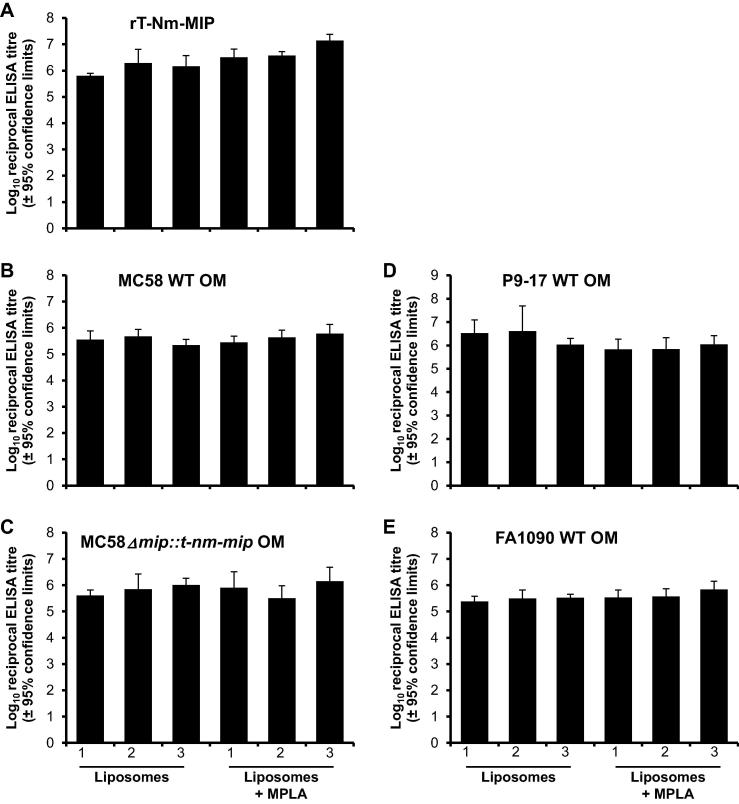
Enzyme-Linked ImmunoSorbent Assays (ELISA). Antisera from individual mice immunised with M2 rT-Nm-MIP-Liposomes and M2 rT-Nm-MIP-Liposomes + MPLA (three independent immunizations each) were reacted against (A) pure M2 rT-Nm-MIP protein, wild-type (WT), (B) MC58 OM, (D) P9-17 OM and (E) FA1090 OM preparations and (C) complemented MC58Δm*ip::t-nm-mip* OM preparation. The columns represent the geometric mean reciprocal ELISA titres (n = 5 animals per group) and the error bars represent the 95% confidence limits. No significant reactivity with pure M2 rT-Nm-MIP protein, WT or complemented OM was observed with sera from sham-immunised animals or with normal mouse serum (NMS) (absorbance values OD_450_nm < 0.1 for serum dilutions of 1/10). All of the test and control sera showed no significant reactivity (*i.e.* absorbance values OD_450_nm < 0.1 for serum dilutions of 1/10) against MC58Δm*ip*, P9-17Δm*ip* or FA1090Δm*ip* OM (data not shown).

The batch consistency of M2 rT-Nm-MIP-liposome (n = 3) and M2 rT-Nm-MIP-liposome + MPLA (n = 3) vaccines was assessed initially by testing individual antisera in ELISA against the immunizing protein, homologous MC58 OM, and OM from a MC58 *mip* knock-out strain that was engineered to constitutively express the *mip* gene fragment encoding the truncated protein without the 6 × HIS tag (AA22-143). High titres of antibodies, reactive with homologous immunizing protein, were induced by rT-Nm-MIP in all six liposomal preparations (Fig. 2A), and no significant differences between the mean antibody titres was observed (mean ∼2.6 × 10^6^, range 0.6 × 10^6^–1.2 × 10^7^, P > 0.05). These antibodies also reacted with homologous OM (Fig. 2B) and the engineered rT-Nm-MIP-OM (Fig. 2C), with similar titres observed (∼0.37–0.67 × 10^6^, P > 0.05) with all the immunizing preparations tested against both OM preparations. Notably, antisera to all the rT-Nm-MIP-liposome and rT-Nm-MIP-liposome + MPLA vaccines showed strong cross-reactivity with *N. gonorrhoeae* P9-17 (Fig. 2D) and FA1090 (Fig. 2E) OM preparations, with similar mean reciprocal titres (0.4–1.4 × 10^6^), not significantly different (P > 0.05) from those observed on meningococcal OM. No significant reactivity (P > 0.05) was observed for any antiserum against MC58Δm*ip*, P9-17Δm*ip* or FA1090Δm*ip* OM preparations (data not shown).

**Fig. 3:**
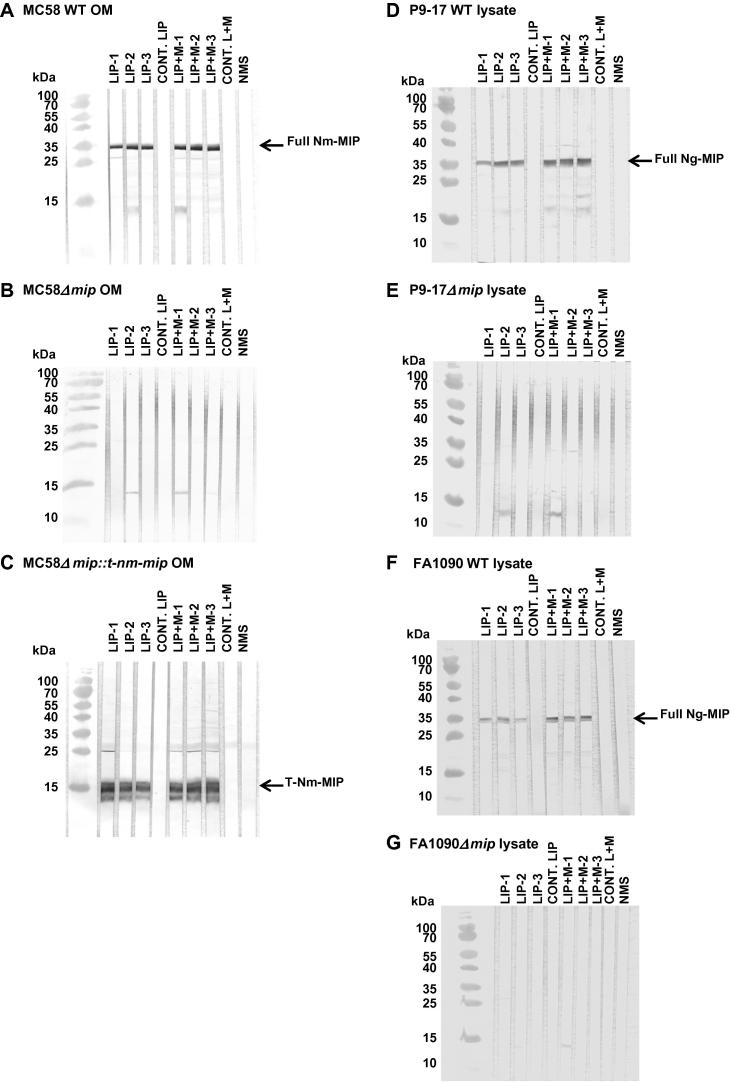
Western immunoblotting of wild-type (WT) and mutant meningococcal OM preparations and gonococcal whole lysates. Pooled murine antisera (1/100 dilution; n = 5 animals) raised against purified M2 rT-Nm-MIP-Liposomes and M2 rT-Nm-MIP-Liposomes + MPLA (three independent immunizations each) were reacted against MC58 WT, Δm*ip* and *Δmip::t-nm-mip* OM preparations (10 µg), or against P9-17 and FA1090 WT and *Δmip* whole lysates (15 µg), in western blot. MIP protein was recognised as a single band of *Mr* ∼ 29 kDa in (A) MC58 WT OM preparation and (D) P9-17 and (F) FA1090 WT lysates (identified by the arrow). (C) Truncated Nm-MIP protein was recognised as a single band of *Mr* ∼ 14 kDa in MC58 complemented OM preparation (identified by the arrow). No significant reactivity was observed with (B) MC58Δm*ip* OM preparation, (E) P9-17Δm*ip* or (G) FA1090Δm*ip* whole lysates with any of the sera tested. All sham immunisation sera and NMS were non-reactive.

The specificity of the immune response against rT-Nm-MIP protein was demonstrated by western blotting. Pooled antisera of the independent batches of rT-Nm-MIP-liposomes and rT-Nm-MIP-liposomes + MPLA vaccines showed strong and similar reactivity with a single band of molecular mass (*Mr)* ∼29 kDa on MC58 OM (Fig. 3A) or ∼14 kDa (Fig. 3C) on MC58Δm*ip::t-nm-mip* OM. Specificity was confirmed by the lack of reactivity of any of the antisera with MC58Δm*ip* mutant OM preparation (Fig. 3B). These antisera showed cross-reactivity with gonococci, specifically recognising a single band of *Mr* ∼ 29 kDa on western blots of P9-17 (Fig. 3D) and FA1090 (Fig. 3F) lysates. Specificity was confirmed by the lack of reactivity of any of the antisera with P9-17Δm*ip* (Fig. 3E) or FA1090Δm*ip* (Fig. 3G) mutant strains.

### 3.3 Antibodies to rT-Nm-MIP recognize MIP protein expressed on the surface of live meningococci and gonococci

**Fig. 4:**
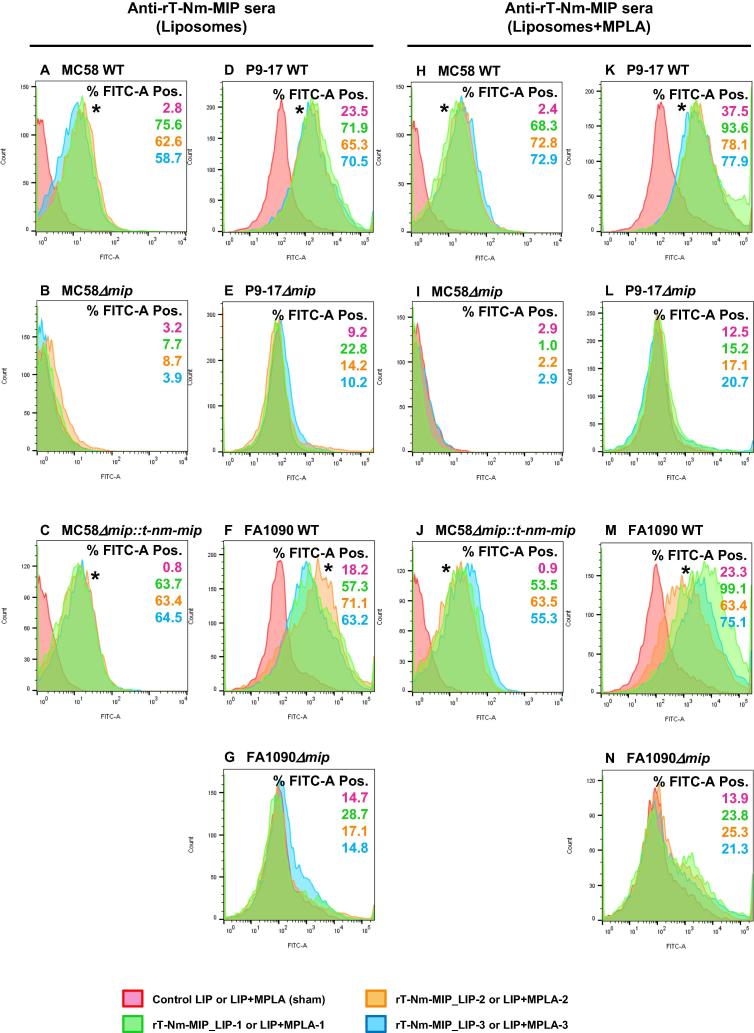
Flow cytometry analysis on wild-type (WT) and mutant meningococcal and gonococcal strains. Murine antisera from three independent immunizations with M2 rT-Nm-MIP-Liposomes were reacted against MIP on the surface of (A) MC58, (D) P9-17 and (F) FA1090 WT strains, or against (C) rT-Nm-MIP on MC58 engineered to express constitutively the truncated protein. Murine antisera from three independent immunizations against M2 rT-Nm-MIP-Liposomes + MPLA were reacted against MIP on the surface of (H) MC58, (K) P9-17 and (M) FA1090 WT strains, or against (J) rT-Nm-MIP on MC58 engineered to express constitutively the truncated protein. The pink area shows no reactivity of the meningococcal or gonococcal strains with murine sham-immunised serum (1/10). The green, orange and blue areas show the significant FACS reactivity of murine antisera (1/10) to M2 rT-Nm-MIP-Liposomes (batches 1, 2 and 3) or M2 rT-Nm-MIP-Liposomes + MPLA (batches 1, 2 and 3) respectively, with MC58, P9-17 and FA1090 WT strains, and with complemented MC58 strain. All antisera were non-reactive against the corresponding MC58 (B, I), P9-17 (E, L) or FA1090 (G, N) *nm-mip* isogenic knock-out strains. The numbers within each panel refer to the percentage of bacterial populations that were FITC-positive. The asterisks (*) denote the significant (P < 0.05) and right-shifted increases in FITC-fluorescence recorded events, using a two sample *t*-Test to compare mean fluorescence values of test murine antisera against sham-immunised murine sera. Data are representative of n = 2 experiments. (For interpretation of the references to colour in this figure legend, the reader is referred to the web version of this article.)

Flow cytometry demonstrated that antisera to rT-Nm-MIP-liposomes and rT-Nm-MIP-liposome + MPLA preparations reacted with live wild-type MC58 (Nm-MIP^+^) and complemented (Truncated-Nm-MIP^+^) bacteria, showing significant (P < 0.05) right-shifted increases in FITC-fluorescence recorded events, compared to sham-immunised murine sera (Fig. 4). Antisera cross-reacted similarly with Ng-MIP on the surface of live P9-17 and FA1090 gonococci. Specificity was confirmed by the lack of reactivity (P > 0.05) of all murine post-immune sera with MC58Δm*ip*, P9-17Δm*ip* or FA1090Δm*ip* strains (Fig. 4).

### 3.4 Murine antibodies to rT-Nm-MIP are bactericidal for meningococci and gonococci

Using the HC-SBA, murine antisera raised to M2 rT-Nm-MIP were bactericidal for the homologous strains MC58 and MC168 with median titres of 32–64 for the rT-Nm-MIP-liposome preparations and 128–256 for the rT-Nm-MIP-liposome + MPLA preparations (Table 2). Specificity was demonstrated by the inability of antisera to kill the isogenic MC58Δm*ip* strain. Antisera to M2 rT-Nm-MIP also induced complement-mediated killing of MenB strains expressing heterologous M1 protein, with bactericidal titres of 64 with rT-Nm-MIP-liposome preparations and 128–256 with rT-Nm-MIP-liposome + MPLA (Table 2). By contrast, no killing was observed for any of the three MenB strains expressing M6 Nm-MIP protein. Of the two MenB strains that expressed M7 protein tested, one (M16 240169) was killed by antisera to rT-Nm-MIP-lipsomes + MPLA at low levels (titres 4–8), whereas the other (M15 240139) was not killed.

**Table 2:** Bactericidal activity of pooled antisera against rT-Nm-MIP (M2) protein delivered in different liposomes formulations. The titres are expressed as the reciprocal of the highest dilution at which 50% killing was observed. Human complement (HC, 25% [v/v]) or normal human serum (HS, 17% [v/v]) were used as exogenous complement sources for meningococcal and gonococcal strains, respectively. Data are the median values, with the range of values in parentheses, for HC-SBA from 3 to 4 independent measurements of bactericidal activity of all pooled serum samples. Single values denote that the HC-SBA titres from the independent experiments were identical. Significantly high bactericidal activities relative to the corresponding controls are highlighted in red. Sera from sham-immunized mice (controls) and normal mouse serum (NMS) showed no significant bactericidal activity. Strains MS11 and 12CFX_T_003 are serum sensitive. ND, not determined.

**Fig. 5:**
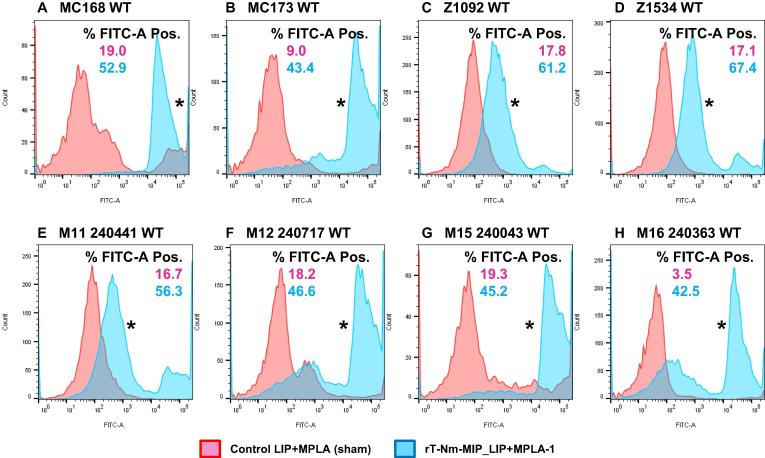
Flow cytometry analysis on wild-type (WT) meningococcal strains expressing M2 Nm-MIP protein. Murine pooled antisera (n = 5 mice) raised against M2 rT-Nm-MIP-Liposomes + MPLA (batch 1) and the corresponding control antisera were reacted against M2 Nm-MIP protein on the surface of (A) MC168 (MenB), (B) MC173 (MenC), (C) Z1092 (MenA), (D) Z1534 (MenA), (E) M11 240,441 (MenW), (F) M12 240,717 (MenY), (G) M15 240,043 (MenY), and (H) M16 240,363 (MenY) WT strains. The pink area shows no reactivity of murine sham-immunised serum (1/10) with any of the meningococcal WT strains tested. The blue area shows the significant FACS reactivity of murine antisera (1/10) to M2 rT-Nm-MIP-Liposomes + MPLA (batch 1) with all meningococcal WT strains tested. The numbers within each panel refer to the percentage of bacterial populations that were FITC-positive. The asterisks (*) denote the significant (P < 0.05) and right-shifted increases in FITC-fluorescence recorded events, using a two sample *t*-Test to compare mean fluorescence values of test murine antisera against sham-immunised murine sera. Data are representative of n = 2 experiments. (For interpretation of the references to colour in this figure legend, the reader is referred to the web version of this article.)

For meningococci expressing homologous M2 protein but different serogroup capsule, significant killing was observed against MenC and MenW bacteria (Table 2). For MenC, rT-Nm-MIP-liposomes + MPLA antisera were bactericidal with a titre of 16; for MenW, rT-Nm-MIP-liposomes and rT-Nm-MIP-liposomes + MPLA antisera showed bactericidal titres of 8–16. By contrast, no significant bactericidal effect was observed for MenA or MenY bacteria (Table 2). It is possible that variation in surface expression of M2 protein accounted for the differences in bactericidal activity observed between these different serogroup meningococci. However, FACS analyses showed no significant differences (P > 0.05) of surface expression of M2 protein in the MenA, MenB, MenC, MenW and MenY isolates (Fig. 5).

Using HS-SBA, antisera to M2 rT-Nm-MIP-liposomes and M2 rT-Nm-MIP-liposomes + MPLA also showed significant bactericidal activity against gonococcal strain P9-17 that expressed M35 protein, with titres of 64–128 and 64–512, respectively (Table 2). Specificity was shown by the inability of any antisera to kill the isogenic P9-17Δm*ip* strain (Table 2). Murine antisera also killed one strain expressing M10 protein, 12CFX_T_003 (titres 8–16), but not another, MS11. No killing was observed for strain FA1090, which expressed the M8 protein (Table 2).

### 3.5 Expression of a truncated-Nm-MIP protein in the meningococcal OM

An alternative strategy for presentation of truncated Nm-MIP protein was attempted. A MC58 Δm*ip* strain was designed to over-express constitutively M2 T-Nm-MIP protein under the transcriptional control of a hybrid, strong *PorA/NadA* promoter, and lipooligosaccharide (LOS)-reduced OM then produced. The *lpxA* gene could not be knocked-out in a MC58Δm*ip* strain or the complemented MC58Δm*ip::t-nm-mip* strain, using either piliated and non-piliated (non-naturally competent) *N. meningitidis* transformation methods [33]. Instead, LOS was extracted from wild-type MC58, MC58Δm*ip* and MC58Δm*ip::t-nm-mip* bacteria with Na-DOC, and mice were immunized with all three native OM and Na-DOC OM preparations (Supplementary Fig. 5). However, western blotting (Supplementary Fig. 6) and mass spectrometry analysis (data not shown) of the native OM, Na-DOC OM and all corresponding washes throughout the extraction process, demonstrated high solubility of full-length mature and truncated Nm-MIP proteins in Na-DOC that resulted in a significant loss of target antigen from within the OM.

## 4 Discussion

The major findings from the current study were: (1) different batches of rT-Nm-MIP protein in liposomes and liposomes + MPLA showed no significant batch-to-batch variation in antigenicity; (2) the antibodies induced reacted specifically with Nm-MIP and Ng-MIP in their OM preparations and bound equally and specifically to live meningococcal and gonococcal cell surfaces; (3) use of HC/HS-SBA demonstrated that antisera to rT-Nm-MIP preparations killed meningococci and were cross-protective against gonococci, but there was significant divergence in bactericidal responses between different strains; (4) meningococcal OM were engineered to express T–Nm-MIP constitutively and at high levels as an alternative to recombinant protein production, but the use of Na-DOC for LOS extraction was contra-indicated.

Previously, we demonstrated using the BRC-SBA that antisera to M2 rT-Nm-MIP (Type I) protein were bactericidal against the (i) homologous MenB strain, (ii) heterologous MenB strains expressing M1 (Type II) and M6 (Type III) proteins and (iii) heterologous MenA, MenC, MenW and MenY bacteria expressing homologous M2 Nm-MIP [16]. However, the HC-SBA assays used in the current study demonstrated that the bactericidal responses and the levels of cross-protection determined with the previous BRC-SBA were over-estimates. Thus, no cross-protective bactericidal activity was observed against M6-expressing strains, or against MenA or MenY bacteria expressing homologous M2 Nm-MIP protein. Against strains expressing M7 (Type IV) protein, the bactericidal activity levels were marginal. For the gonococcal strains tested, bactericidal activity was observed for P9-17 (M35 Ng-MIP) and one strain 12CFX_T_003 (M10 Ng-MIP) but not for a strain expressing M8 Ng-MIP.

The lower bactericidal responses observed with the HC-SBA are consistent with the use of the more stringent complement source [20], [21], [22]. Although the N-terminal region of MIP is well-conserved, we examined whether the specific amino acid residue differences influenced bactericidal activity. Marginal levels of killing were observed for meningococci expressing M7 protein, which showed a deletion (amino acid residues 28–31) and a Ser39Ala change, compared to M2 protein (Fig. 4). However, these are also the only two changes in N-terminal M1 protein, and meningococci expressing this type were killed, suggesting that these changes did not influence bactericidal activity with the M7-expressing strain. By contrast, Asp123 residue, which is conserved in all of the protein types, is changed to a Glu only in the M7 type protein and therefore may be important for bactericidal activity. Meningococci expressing M6 protein type were also not killed. In this protein, Ala140 and Asp142, which are conserved in the N-terminal domain of all of the other protein types, is changed to a Gly and Glu residue, respectively. Therefore, it is possible that the Ala140 and Asp142 residues are important for inducing bactericidal antibodies.

We examined also the differences in amino acid sequences in the gonococcal MIP protein types, compared to meningococcal M2. In our study, gonococcal isolates expressing both M10 and M35 proteins were killed. The N-terminal domains of both proteins were identical with the only three changes being Ser39Ala, Ser49Gly and Glu83Asp (Fig. 4). Surprisingly, gonococci expressing M8 protein, which also has the Ser39Ala and Glu83Asp substitutions, was not killed. Taken together, these observations suggest that these three amino acid residues are not important for the bactericidal response. It is possible that PorinB-mediated serum resistance imparted by binding of complement proteins and factor H [34] prevented the bactericidal activity of M2 rT-Nm-MIP antiserum, instead of these amino acid sequence differences. However, a limitation of the current study is that significantly larger numbers of isolates should be tested to confirm these findings.

Unexpectedly, there was variation in bactericidal activity of antisera to M2 rT-Nm-MIP tested against M2-expressing meningococci of different serogroups. Differential surface expression of Nm-MIP was not a factor (Fig. 5), suggesting several hypotheses: i) MenA and MenY capsules could hinder antibody binding to surface-exposed OM antigens, perhaps associated with capsule structure and density and ii) MIP could be masked by other surface molecules, *e.g.* other OM proteins and/or different terminal extensions of lipooligosaccharide such as the presence of phosphoethanolamine, enhancing serum-resistance [34].

The most effective vaccine formulation was rT-Nm-MIP- liposomes + MPLA, which increased bactericidal titres compared with rT-Nm-MIP-liposomes, albeit by only a factor of 2–4-fold. Several strategies could be considered for improving the immune response to the truncated protein. These would include testing a broader range of adjuvants and using methods to increase antigen density, *e.g.* by generating fusion proteins, in a manner described with Bexsero antigens [35] or by using virus-like particles [36] to present multiple truncated MIP sequences.

A recent epidemiological study suggested that vaccination with the MeNZB OMV vaccine in New Zealand was associated with statistically significant reduction in the rates of gonorrhoea diagnosis, with an estimated effectiveness of the vaccine against gonorrhoea of 31% [37]. This important observation suggests that meningococcal and gonococcal OM probably share common antigens, including MIP [13], which may contribute to cross-protection. Therefore, engineering Nm-OMV vaccine(s) to express MIP and other proteins that cover the majority of meningococci and gonococci circulating in the populations, is a strategy that could be investigated. In the current study, we were able to engineer successfully the Nm-OM to express M2 Truncated –Nm-MIP constitutively and at high levels. However, LOS-extraction with Na-DOC was contra-indicated, as it led to loss of the majority of T-Nm-MIP from the OM. Thus, future studies to engineer the OM to contain T-Nm-MIP should avoid the use of detergents and consider other genetic approaches to detoxify native LOS [38].

## 5 Conclusion

*Neisseria* MIP proteins are potential targets for drug therapies during infection [15] and for the development of prophylactic vaccines [13]. Our data demonstrate the reproducibility of independent vaccines batches for generating bactericidal antibodies against a panel of homologous and heterologous MIP Type and serogroup meningococci, and cross-reactive with some gonococcal strains. The data suggest that the vaccine potential of truncated Ng-MIP proteins should also be examined and that a multi-component vaccine containing a select number of Nm- and Ng-MIP type proteins would be required to provide broad coverage of both pathogens.

## Appendix A Supplementary material

Supplementary Fig. 1 SDS PAGE gel of purified recombinant C-terminal Truncated M2 Nm-MIP protein (rT-Nm-MIP). M2 rT-Nm-MIP protein (*Mr* ∼ 14 kDa) was expressed as recombinant mature soluble protein and purified under native conditions.

Supplementary Fig. 2 Clustal alignment of the non-redundant translated amino acid sequences for MIP proteins corresponding to known alleles found in meningococcal and gonococcal isolates in the pubmlst.org/*Neisseria* database. PubMLST database (https://pubmlst.org/bigsdb?db=pubmlst_neisseria_isolates) was accessed January 2018. Amino acid sequence alignments were generated using Clustal Omega (http://www.ebi.ac.uk/Tools/msa/clustalo/). Nm, meningococcus; Ng, gonococcus. * (asterisk) denotes fully conserved amino acid residue; : (colon) indicates conservation between groups of strongly similar properties; . (period) denotes conservation between groups of weakly similar properties.

Supplementary Fig. 4 Comparison of M2 Nm-MIP (Type I) to M1, M6, M7 Nm-MIP (Types II-IV) and M8, M10 and M35 Ng-MIP proteins. Percentage of identity of M1, M6, M7 Nm-MIP proteins (Types II-IV) and M8, M10 and M35 Ng-MIP proteins to M2 Nm-MIP (Type I) N-terminal domain (amino acid residues 1-142) and C-terminal domain (amino acid residues 143-272) are specified. M2 Nm-MIP leader sequence (amino acid residues 1-22) is coloured in black and the FKBP domain is striped (amino acid residues 166-252).

Supplementary Fig. 5 Analysis of MC58 wild-type (WT), MC58*mip* and MC58*mip::t-nm-mip* OM preparations. (A) OM preparations (20 µg) of MC58 WT, *mip* knock-out and complemented with rT-Nm-MIP strains were reacted with polyclonal rabbit sera (1/400 dilution) raised against full length M2 rNm-MIP Type I [17] in western immunoblotting. Full length WT and truncated Nm-MIP proteins were recognised as a single band of *Mr* ∼ 29 kDa and ∼14 kDa, respectively (identified by the arrow). MIP was not detected in the MC58*mip* OM preparation. (B) SDS-PAGE of 20 µg of each OM preparation.

Supplementary Fig. 6 Western immunobloting of murine antisera to engineered meningococci native and Na-DOC OM. Groups of five BALB/c mice were immunized with three doses of MC58 wild-type (WT) native OM, MC58Δ*mip* OM and complemented MC58Δ*mip::t-nm-mip* OM and the corresponding Na-DOC OM preparations (20 µg/mouse) on days 0, 14, and 28. Groups of five mice were also sham immunized. Pooled murine antisera (1/100 dilution; n = 5 animals) raised against the native and Na-DOC OM preparations were reacted against purified recombinant full length M2 Nm-MIP protein in western blot. rNm-MIP protein was recognised as a single, strong band of *Mr* ∼ 33 kDa with anti-MC58 WT native OM sera. Lower reactivity was observed with antisera to MC58 Na-DOC OM, complemented MC58 OM and Na-DOC OM. No significant reactivity was observed with antisera raised against MC58*mip* OM preparation. Sham immunisation sera were non-reactive.
