# Supplementary material for: Immunization with recombinant truncated Neisseria meningitidis-Macrophage Infectivity Potentiator (rT-Nm-MIP) protein induces murine antibodies that are cross-reactive and bactericidal for Neisseria gonorrhoeae
Source: Vaccine. 2018 Jun 22;36(27):3926–36. doi: 10.1016/j.vaccine.2018.05.069 (PMC6018565; doi:10.1016/j.vaccine.2018.05.069)
Supplement: Supplementary Fig. 1 — SDS PAGE gel of purified recombinant C-terminal Truncated M2 Nm-MIP protein (rT-Nm-MIP). M2 rT-Nm-MIP protein (Mr ∼ 14 kDa) was expressed as recombinant mature soluble protein and purified under native conditions. [file mmc1.pptx]

## Slide 1
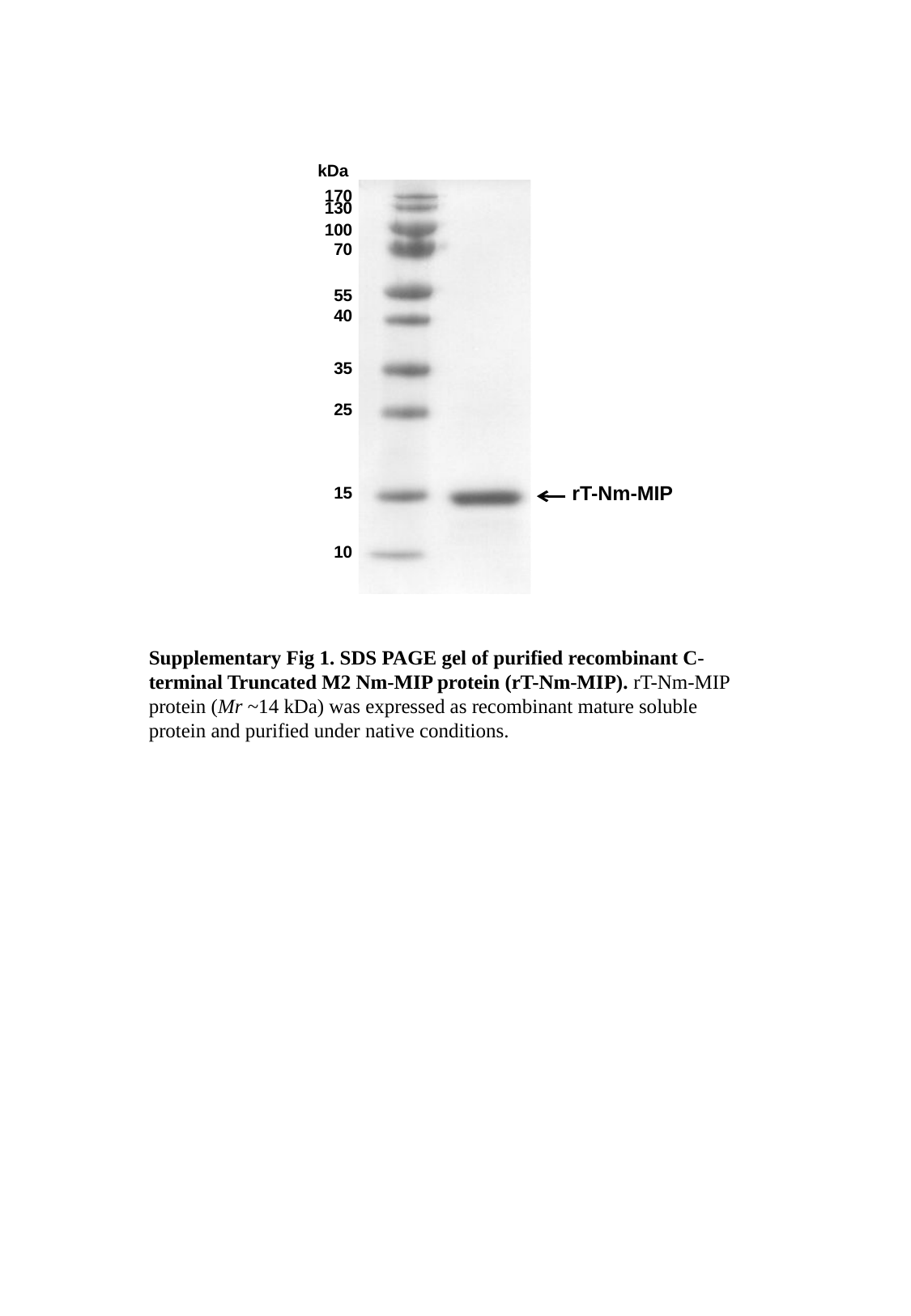

kDa
170
130
100
70
55
40
35
25
rT-Nm-MIP
15
10
Supplementary Fig 1. SDS PAGE gel of purified recombinant C-terminal Truncated M2 Nm-MIP protein (rT-Nm-MIP). rT-Nm-MIP protein (Mr ~14 kDa) was expressed as recombinant mature soluble protein and purified under native conditions.
