# Supplementary material for: Immunization with recombinant truncated Neisseria meningitidis-Macrophage Infectivity Potentiator (rT-Nm-MIP) protein induces murine antibodies that are cross-reactive and bactericidal for Neisseria gonorrhoeae
Source: Vaccine. 2018 Jun 22;36(27):3926–36. doi: 10.1016/j.vaccine.2018.05.069 (PMC6018565; doi:10.1016/j.vaccine.2018.05.069)
Supplement: Supplementary Fig. 2 — Clustal alignment of the non-redundant translated amino acid sequences for MIP proteins corresponding to known alleles found in meningococcal and gonococcal isolates in the pubmlst.org/Neisseria database. PubMLST database (https://pubmlst.org/bigsdb?db=pubmlst_neisseria_isolates) was accessed January 2018. Amino acid sequence alignments were generated using Clustal Omega (http://www.ebi.ac.uk/Tools/msa/clustalo/). Nm, meningococcus; Ng, gonococcus. * (asterisk) denotes fully conserved amino acid residue; : (colon) indicates conservation between groups of strongly similar properties; . (period) denotes conservation between groups of weakly similar properties. [file mmc2.docx]

Nm_113 MNKIFKISALTLAATLALSACGKKENVPAS-SASEPAAASAAQGDTSSIGNTMQQASYAM 59

Nm_229 MNKIFKISALTLAATLALSACGKKEAAPA--SASEPAAASAAQGDTSSIGSTMQQASYAM 58

Nm_27 MNTIFKISALTLSAALALSACGKKEAASASASASEPAAASAAQGDTSSIGSTMQQASYAM 60

Nm_319 MNTIFKISALTLSAALALSACGKKEAA------SEPAAASAAQGDTSSIGSTMQQASYAM 54

Ng_200 MNTIFKISALTLSAALALSACGKKEAAPA--SASEPS---AAQGDTSSIGSTMQQASYAM 55

Ng_56 MNTIFKISALTLSAALALSACGKKEAAPA--PASEPAAASAAQGDTFSIGGTMQQASYAM 58

Ng_208 MNTIFKISALTLSAALALSACGKKEAAPA--PASEPAAASAAQGDTSSIGGTMQQASYAM 58

Ng_207 MNTIFKISALTLSAALALSACGKKEAVPA--PASEPAAASAAQGDTSSIGSTMQQASYAM 58

Ng_203 MNTIFKISALTLSAALALSACGKKEAAPA--PASEPAAASAAQGDTSSIGSTMQQASYAM 58

Ng_204 MNTIFKISALTLSAALALSACGKKEAAPA--SASEPAAASAAQGDTSSIGGTMQQASYAM 58

Ng_137 MNTIFKISALTLSAALALSACGKKEAAPA--SASEPAAASAAQGDTSSIGGTMQQASYAM 58

Ng_225 MNTIFKISALTLSAALALSACGKKEAAPA--SASEPAAASAAQGDTSSIGSTMQQASYAM 58

Ng_8 MNTIFKISALTLSAALALSACGKKEAAPA--SASEPAAASAAQGDTSSIGSTMQQASYAM 58

Ng_35 MNTIFKISALTLSAALALSACGKKEAAPA--SASEPAAASAAQGDTSSIGGTMQQASYAM 58

Ng_209 MNTIFKISALTLSAALALSACGKKEAAPA--SASEPAAASAAQGDTSSIGGTMQQASYAM 58

Ng_140 MNTIFKISALTLSAALALSACGKKEAAPA--SASEPAAASAAQGDTSSIGSTMQQASYAM 58

Ng_202 MNTIFKISALTLSAALALSACGKKEAAPA--PASEPAAASAAQGDTSSIGGTMQQASYAM 58

Ng_409 MNTIFKISALTLSAALALSACGKKEAAPA--SASEPAAASAAQGDTSSIGGTMQQASYAM 58

Ng/Nm_10 MNTIFKISALTLSAALALSACGKKEAAPA--SASEPAAASAAQGDTSSIGGTMQQASYAM 58

Ng_211 MNTIFKISALTLSAALALSACGKKEAAPA--PASEPAAASAAQGDTSSIGSTMQQASYAM 58

Ng_205 MNTIFKISALTLSAALALSACGKKEAAPA--PASEPAAASAAQGDTSSIGSTMQQASYAM 58

Nm_324 MNTIFKISALTLAAALALSACGKKEAAPAA-SASEPAAASAAQGDTSSIGSTMQQASYAM 59

Nm_15 MNTIFKISALTLSAALALSACGKKEAAPAA-TASEPAAASAAQGDTSSIGSTMQQASYAM 59

Nm_116 MNTIFKISALTLSAALALSACGKKEAAPAS--ASEPAAASSAQGDTSSIGSTMQQASYAM 58

Nm_295 MNTIFKISALTLSAALALSACGKKEAA------SEPAAASAAQGDTSSIGSTMQQASYAM 54

Nm_6 MNTIFKISALTLSAALALSACGKKEAAPAS--ASEPAAASSAQGDTSSIGSTMQQASYAM 58

Nm_127 MNTIFKISALTLSAALALSACGKKEAASAS--ASEPAAASAAQGDTSSIGSTMQQASYAM 58

Nm_130 MNTIFKISALTLSAALALSACGKKEAA------SEPAAASAAQGDTSSIGSTMQQASYAM 54

Nm_107 MNTIFKISALTLSAALALSACGKKEAA------SEPAAASAAQGDTSSIGSTMQQASYAM 54

Nm_419 MNTIFKISALTLSAALALSACGKKEAA------SEPTAASAAQGDTSSIGSTMQQASYAM 54

Nm_366 MNTIFKISALTLSAALALSACGKKEAA------SEPAAASAAQGDTSSIGSTMQQASYAM 54

Nm_302 MNTIFKISALTLSAALALSACGKKEAA------SEPAAASAAQGDTSSIGSTMQQASYAM 54

Nm_294 MNTIFKISALTLSAALALSACGKKEAA------SEPAAASAAQGDTSSIGSTMQQASYAM 54

Nm_287 MNTIFKISALTLSAALALSACGKKEAA------SEPAAASAAQGDTSSIGSTMQQASYAM 54

Nm_259 MNTIFKISALTLSAALALSACGKKEAA------SEPAAASAAQGDTSSIGSTMQQASYAM 54

Nm_247 MNTIFKISALTLSAALALSACGKKEAA------SEPAAASSAQGDTSSIGSTMQQASYAM 54

Nm_240 MNTIFKISALTLSAALALSACGKKEAA------SEPAAASAAQGDTSSIGSTMQQASYAM 54

Nm_189 MNTIFKISALTLSAALALSACGKKEAA------SEPAAASAAQGDTSSIGSTMQQASYAM 54

Nm_145 MNTIFKISALTLSAALALSACGKKEAA------SEPAAASAAQGDTSSIGSTMQQASYAM 54

Nm_106 MNTIFKISALTLSAALALSACGKKEAA------SEPAAASAAQGDTSSIGSTMQQASYAM 54

Nm-105 MNTIFKISALTLSAALALSACGKKEAA------SEPAAASAAQGDTSSIGSTMQQASYAM 54

Nm_222 MNTIFKISALTLSAALALSACGKKEAA------SEPAAASAAQGDTSSIGSTMQQASYAM 54

Nm_215 MNTIFKISALTLSAALALSACGKKEAA------SEPAAASAAQGDTSSIGSTMQQASYAM 54

Nm_1 MNTIFKISALTLSAALALSACGKKEAA------SEPAAASAAQGDTSSIGSTMQQASYAM 54

Nm_7 MNTIFKISALTLSAALALSACGKKEAA------SEPAAASAAQGDTSSIGSTMQQASYAM 54

Nm_22 MNTIFKISALTLSAALALSACGKKEAA------SEPAAASAAQGDTSSIGSTMQQASYAM 54

Nm_141 MNTIFKISALTLSAALALSACGKKEAAPAS--ASEPAAASSAQGDTSSIGSTMQQASYAM 58

Nm_374 MNTIFKISALTLSAALALSACGKKEAAPAS--ASEPAAASSAQGDTSSIGSTMQQASYAM 58

Nm_24 MNKIFKISTLTLAATLALSACGKKEAAPAS--ASEPAAASAAQGDTSSIGSTMQQASYAM 58

Nm_3 MNTIFKISALTLSAALALSACGKKEAAPAS--ASEPAAASSAQGDTSSIGSTMQQASYAM 58

Ng/Nm_63 MNTIFKISALTLSAALALSACGKKEAAPAS--ASEPAAASSAQGDTSSIGSTMQQASYAM 58

Nm_265 MNTIFKISALTLSAALALSACGKKEAAPAS--ASEPAAASSAQGDTSSIGSTMQQASYAM 58

Nm_486 MNTIFKISALTLSAALALSACGKKEAAPAS--ASEPAAASSAQGDTSSIGSTMQQASYAM 58

Nm_483 MNTIFKISALTLSAALALSACGKKEAAPAS--ASEPAAASSAQGDTSSIGSTMQQASYAM 58

Nm_466 MNTIFKISALTLSAALALSACGKKEAAPAS--ASEPAAASSAQGDTSSIGSTMQQASYAM 58

Nm_465 MNTIFKISALTLSAALALSACGKKEAAPAS--ASEPAAASSAQGDTSSIGSTMQQASYAM 58

Nm_406 MNTIFKISALTLSAALALSACGKKEAAPAS--ASEPAAASSAQGDTSSIGSTMQQASYAM 58

Nm_405 MNTIFKISALTLSAALALSACGKKEAASAS--ASEPAAASSAQGDTSSIGSTMQQASYAM 58

Nm_367 MNTIFKISALTLSAALALSACGKKEAAPAS--ASEPAAASSAQGDTSSIGSTMQQASYAM 58

Nm_365 MNTIFKISALTLSAALALSACGKKEAAPAS--ASEPAAASSAQGDTSSIGSTMQQASYAM 58

Nm_331 MNTIFKISALTLSAALALSACGKKEAAPAS--ASEPAAASSAQGDTSSIGSTMQQASYAM 58

Nm_320 MNTIFKISALTLSAALAFSACGKKEAAPAS--ASEPAAASSAQGDTSSIGSTMQQASYAM 58

Nm_301 MNTIFKISALTLSAALALSACGKKEAAPAS--ASEPAAASAAQGDTSSIGSTMQQASYAM 58

Nm_197 MNTIFKISALTLSAALALSACGKKEAAPAS--ASEPAAASSAQGDTSSIGSTMQQASYAM 58

Nm_299 MNTIFKISALTLSAALALSACGKKEAAPAS--ASEPAAASSAQGDTSSIGSTMQQASYAM 58

Nm_290 MNTIFKISALTLSAALALSACGKKEAAPAS--ASEPAAASSAQGDTSSIGSTMQQASYAM 58

Nm_257 MNTIFKISALTLSAALALSACGKKEAAPAS--ASEPAAASSAQGDTSSIGSTMQQASYAM 58

Nm_155 MNTIFKISALTLSAALALSACGKKEAAPAS--ASEPAAASSAQGDTSSIGSTMQQASYAM 58

Nm_152 MNTIFKISALTLSAALVLSACGKKEAAPAS--ASEPAAASSAQGDTSSIGSTMQQASYAM 58

Nm_148 MNTIFKISALTLSAALALSACGKKEAAPAS--ASEPAAASSAQGDTSSIGSTMQQASYAM 58

Nm_147 MNTIFKISALTLSAALALSACGKKEAAPAS--ASEPAAASSAQGDTSSIGSTMQQASYAM 58

Nm_146 MNTIFKISALTLSAALALSACGKKEAAPAS--ASEPAAASSAQGDTSSIGSTMQQASYAM 58

Nm_132 MNTIFKISALTLSAALALSACGKKEAAPAS--ASEPAAASAAQGDTSSIGSTMQQASYAM 58

Nm_125 MNTIFKISALTLSAALALSACGKKEAAPAS--ASEPAAASSAQGDTSSIGSTMQQASYAM 58

Nm_118 MNTIFKISALTLSAALALSACGKKEAAPAS--ASEPAAASAAQGDTSSIGSTMQQASYAM 58

Nm_114 MNTIFKISALTLSAALALSACGKKEAAPAS--ASEPAAASSAQGDTSSIGSTMQQASYAM 58

Nm_96 MNTIFKISALTLSAALALSACGKKEAAPAS--ASEPAAASSAQGDTSSIGSTMQQASYAM 58

Nm_112 MNTIFKISALTLSAALALSACGKKEAAPAS--ASEPAAASSAQGDTSSIGSTMQQASYAM 58

Nm_110 MNTIFKISALTLSAALALSACGKKEAAPAS--ASEPAAASSAQGDTSSIGSTMQQASYAM 58

Nm_94 MNTIFKISALTLSAALALSACGKKEAAPAS--TSEPAAASSAQGDTSSIGSTMQQASYAM 58

Nm_109 MNTIFKISALTLSAALALSACGKKEATPAS--ASEPAAASSAQGDTSSIGSTMQQASYAM 58

Nm_5 MNTIFKISALTLSAALALSACGKKEAAPAS--ASEPAAASSAQDDTSSIGSTMQQASYAM 58

Nm_74 MNTIFKISALTLSAALALSACGKKEAAPAS--ASEPAAASSAQGDTSSIGSTMQQASYAM 58

Nm_69 MNTIFKISALTLSAALALSACGKKEAAPAS--ASEPAAASSAQGDTSSIGSTMQQASYAM 58

Nm_219 MNTIFKISALTLSAALALSACGKKEAAPAS--ASEPAAASSAQGDTSSIGSTMQQASYAM 58

Nm_217 MNTIFKISALTLSAALALSACGKKEAAPAS--ASEPAAASAAQGDTSSIGSTMQQASYAM 58

Nm_216 MNTIFKISALTLSAALALSACGKKEAAPAS--ASEPAAASSAQGDTSSIGSTMQQASYAM 58

Nm_31 MNTIFKISALTLSAALALSACGKKEAAPAS--ASEPAAASAAQGDTSSIGSTMQQASYAM 58

Nm_26 MNTIFKISALTLSAALALSACGKKEAAPAS--ASEPAAASSAQGDTSSIGSTMQQASYAM 58

Nm_4 MNTIFKISALTLSAALALSACGKKEAAPAS--ASEPAAASSAQGDTSSIGSTMQQASYAM 58

Nm_11 MNTIFKISALTLSAALALSACGKKEAAPAS--ASEPAAASSAQGDTSSIGSTMQQASYAM 58

**.*****:***:*:*.:******* . ***: :**.** ***.*********

Nm_113 GVDIGRSLKQMKEQGAEIDLKVFTEAMQAMYDGKEIKMTEEQAQEVMMKFLQEQQAKAVE 119

Nm_229 GVDIGRSLKQMKEQGAEIDLKVFTEAMQAVYDGKEIKMTEEQAQEVMMKFLQEQQAKAVE 118

Nm_27 GVDIGRSLKQMKEQGAEIDLKVFTEAMQAVYDGKEIKMTEEQAQEVMMKFLQEQQAKAVE 120

Nm_319 GVDIGRSLKQMKEQGAEIDLKVFTEAMQAVYDGKEIKMTEEQAQEVMMKFLQEQQAKAVE 114

Ng_200 GVDIGRSLKQMKEQGAEIDLKVFTDAMQAVYDGKEIKMTEEQAQEVMMKFLQEQQAKAVE 115

Ng_56 GVDIGRSLKQMKEQGAEIDLKVFTDAMQAVYDGKEIKMTEEQAQEVMMKFLQEQQAKAVE 118

Ng_208 GVDIGRSLKQMKEQGAEIDLKVFTDAMQAVYDGKEIKMTEEQAQEVMMKFLQEQQAKAVE 118

Ng_207 GVDIGRSLKQMKEQGAEIDLKVFTDAMQAVYDGKEIKMTEEQAQEVMMKFLQEQQAKAVE 118

Ng_203 GVDIGRSLKQMKEQGAEIDLKVFTDAMQAVYDGKEIKMTEEQAQEVMMKFLQEQQAKAVE 118

Ng_204 GVDIGRSLKQMKEQGAEIDLKVFTDAMQAVYDGKEIKMTEEQAQEVMMKFLQEQQAKAVE 118

Ng_137 GVDIGRSLKQMKEQGAEIDLKVFTDAMQAVYDGKEIKMTEEQAQEVMMKFLQEQQAKAVE 118

Ng_225 GVDIGRSLKQMKEQGAEIDLKVFTDAMQAVYDGKEIKMTEEQAQEVMMKFLQEQQAKAVE 118

Ng_8 GVDIGRSLKQMKEQGAEIDLKVFTDAMQAVYDGKEIKMTEEQAQEVMMKFLQEQQAKAVE 118

Ng_35 GVDIGRSLKQMKEQGAEIDLKVFTDAMQAVYDGKEIKMTEEQAQEVMMKFLQEQQAKAVE 118

Ng_209 GVDIGRSLKQMKEQGAEIDLKVFTDAMQAVYDGKEIKMTEEQVQEVMMKFLQEQQAKAVE 118

Ng_140 GVDIGRSLKQMKEQGAEIDLKVFTDAMQAVYDGKEIKMTEEQAQEVMMKFLQEQQAKAVE 118

Ng_202 GVDIGRSLKQMKEQGAEIDLKVFTDAMQAVYDGKEIKMTEEQAQEVMMKFLQEQQAKAVE 118

Ng_409 GVDIGRSLKQMKEQGAEIDLKVFTDAMQAVYDGKEIKMTEEQAQEVMMKFLQEQQAKAVE 118

Ng/Nm_10 GVDIGRSLKQMKEQGAEIDLKVFTDAMQAVYDGKEIKMTEEQAQEVMMKFLQEQQAKAVE 118

Ng_211 GVDIGRSLKQMKEQGAEIDLKVFTDAMQAVYDGKEIKMTEEQVQEVMMKFLQEQQAKAVE 118

Ng_205 GVDIGRSLKQMKEQGAEIDLKVFTDAMQAVYDGKEIKMTEEQAQEVMMKFLQEQQAKAVE 118

Nm_324 GVDIGRSLKQMKEQGAEIDLKVFTEAMQAVYEGKEIKMTEEQAQEVMMKFLQEQQAKAVE 119

Nm_15 GVDIGRSLKQMKEQGAEIDLKVFTEAMQAVYEGKEIKMTEEQAQEVMMKFLQEQQAKAVE 119

Nm_116 GVDIGRSLKQMKEQGAEIDLKVFTEAMQAVYDGKEIKMTEEQAQEVMMKFLQEQQAKAVE 118

Nm_295 GVDIGRSLKQMKEQGAEIDLKVFTEAMQAVYDGKEIKMTEEQAQEVMMKFLQEQQAKAVE 114

Nm_6 GVDIGRSLKQMKEQGAEIDLKVFTEAMQAVYDGKEIKMTEEQAQEVMMKFLQEQQAKAVE 118

Nm_127 GVDIGRSLKQMKEQGAEIDLKVFTEAMQAVYDGKEIKMTEEQAQEVMMKFLQEQQAKAVE 118

Nm_130 GVDIGRSLKQMKEQGAEIDLKVFTEAMQAVYDGKEIKMTEEQAQEVMMKFLQEQQAKAVE 114

Nm_107 GVDIGRSLKQMKEQGAEIDLKVFTEAMQAVYDGKEIKMTEEQAQEVMMKFLQEQQAKAVE 114

Nm_419 GVDIGRSLKQMKEQGAEIDLKVFTEAMQAVYDGKEIKMTEEQAQEVMMKFLQEQQAKAVE 114

Nm_366 GVDIGRSLKQMKEQGAEIDLKVFTEAMQAVYDGKEIKMTEEQAQEVMMKFLQEQQAKAVE 114

Nm_302 GVDIGRSLKQMKEQGAEIDLKVFTEAMQAVYDGKEIKMTEEQAQEVMMKFLQEQQAKAVE 114

Nm_294 GVDIGRSLKQMKEQGAEIDLKVFTEAMQAVYDGKEIKMTEEQAQEVMMKFLQEQQAKAVE 114

Nm_287 GVDIGRSLKQMKEQGAEIDLKVFTEAMQAVYDGKEIKMTEEQAQEVMMKFLQEQQAKAVE 114

Nm_259 GVDIGRSLKQMKEQGAEIDLKVFTEAMQAVYDGKEIKITEEQAQEVMMKFLQEQQAKAVE 114

Nm_247 GVDIGRSLKQMKEQGAEIDLKVFTEAMQAVYDGKEIKMTEEQAQEVMMKFLQEQQAKAVE 114

Nm_240 GVDIGRSLKQMKEQGAEIDLKVFTEAMQAVYDGKEIKMTEEQAQEVMMKFLQEQQAKAVE 114

Nm_189 GVDIGRSLKQMKEQGAEIDLKVFTEAMQAVYDGKEIKMTEEQAQEVMMKFLQEQQAKAVE 114

Nm_145 GVDIGRSLKQMKEQGAEIDLKVFTEAMQAVYDGKEIKMTEEQAQEVMMKFLQEQQAKAVE 114

Nm_106 GVDIGRSLKQMKEQGAEIDLKVFTEAMQAVYDGKEIKMTEEQAQEVMMKFLQEQQAKAVE 114

Nm-105 GVDIGRSLKQMKEQGAEIDLKVFTEAMQAVYDGKEIKMTEEQAQEVMMKFLKEQQAKAVE 114

Nm_222 GVDIGRSLKQMKEQGAEIDLKVFTEAMQAVYDGKEIKMTEEQAQEVMMKFLQEQQAKAVE 114

Nm_215 GVDIGRSLKQMKEQGAEIDLKVFTEAMQAVYDGKEIKMTEEQAQEVMMKFLQEQQAKAVE 114

Nm_1 GVDIGRSLKQMKEQGAEIDLKVFTEAMQAVYDGKEIKMTEEQAQEVMMKFLQEQQAKAVE 114

Nm_7 GVDIGRSLKQMKEQGAEIDLKVFTEAMQAVYDGKEIKMTEEQAQEVMMKFLQEQQAKAVE 114

Nm_22 GVDIGRSLKQMKEQGAEIDLKVFTEAMQAVYDGKEIKMTEEQAQEVMMKFLQEQQAKAVE 114

Nm_141 GVDIGRSLKQMKEQGAEIDLKVFTEAMQAVYDGKEIKMTEEQAQEVMMKFLQEQQAKAVE 118

Nm_374 GVDIGRSLKQMKEQGAEIDLKVFTEAMQAVYEGKEIKMTEEQAQEVMMKFLQEQQAKAVE 118

Nm_24 GVDIGRSLKQMKEQGAEIDLKVFTEAMQAVYDGKEIKMTEEQAQEVMMKFLQEQQAKAVE 118

Nm_3 GVDIGRSLKQMKEQGAEIDLKVFTEAMQAVYDGKEIKMTEEQAQEVMMKFLQEQRAKAVE 118

Ng/Nm_63 GVDIGRSLKQMKEQGAEIDLKVFTEAMQAVYDGKEIKMTEEQAQEVMMKFLQEQQAKAVE 118

Nm_265 GVDIGRSLKQMKEQGAEIDLKVFTEAMQAVYDGKEIKMTEEQAQEVMMKFLQEQQAKAVE 118

Nm_486 GVDIGRSLKQMKEQGAEIDLKVFTEAMQAVYDGKEIKMTEEQAQEVMMKFLQEQQAKAVE 118

Nm_483 GVDIGRSLKQMKEQGAEIDLKVFTEAMQAVYDGKEIKMTEEQAQEVMMKFLQEQQAKALE 118

Nm_466 GVDIGRSLKQMKEQGAEIDLKVFTEAMQAVYDGKEIKMTEEQAQEVMMKFLQEQQAKAVE 118

Nm_465 GVDIGRSLKQMKEQGAEIDLKVFTEAMQAVYDGKEIKMTEEQAQEVMMKFLQEQQAKAVE 118

Nm_406 GVDIGRSLKQMKEQGAEIDLKVFTEAMQAVYDGKEIKMTEEQAQEVMMKFLQEQQAKAVE 118

Nm_405 GVDIGRSLKQMKEQGAEIDLKVFTEAMQAVYDGKEIKMTEEQAQEVMMKFLQEQQAKAVE 118

Nm_367 GVDIGRSLKQMKEQGAEIDLKVFTEAMQAVYDGKEIKMTEEQAQEVMMKFLQEQQAKAVE 118

Nm_365 GVDIGRSLKQMKEQGAEIDLKVFTEAMQAVYDSKEIKMTEEQAQEVMMKFLQEQQAKAVE 118

Nm_331 GVDIGRSLKQMKEQGAEIDLKVFTEAMQAVYDGKEIKMTEEQAQEVMMKFLQEQQAKAVE 118

Nm_320 GVDIGRSLKQMKEQGAEIDLKVFTEAMQAVYDGKEIKMTEEQAQEVMMKFLQEQQAKAVE 118

Nm_301 GVDIGRSLKQMKEQGAEIDLKVFTEAMQAVYDGKEIKMTEEQAQEVMMKFLQEQQAKAVE 118

Nm_197 GVDIGRSLKQMKEQGAEIDLKVFIEAMQAVYDGKEIKMTEEQAQEVMMKFLQEQQAKAVE 118

Nm_299 GVDIGRSLKQMKEQGAEIDLKVFTEAMQAVYDGKEIKMTEEQAQEVMMKFLQEQQAKAVE 118

Nm_290 GVDIGRSLKQMKEQGAEIDLKVFTEAMQAVYDGKEIKMTEEQAQEVMMKFLQEQQAKAVE 118

Nm_257 GVDIGRSLKQMKEQGAEIDLKVFTEAMQAVYDGKEIKMTEEQAQEVMMKFLQEQQAKAVE 118

Nm_155 GVDIGRSLKQMKEQGAEIDLKVFTEAMQAVYDGKEIKMTEEQAQEVMMKFLQEQQAKAVE 118

Nm_152 GVDIGRSLKQMKEQGAEIDLKVFTEAMQAVYDGKEIKMTEEQAQEVMMKFLQEQQAKAVE 118

Nm_148 GVDIGRSLKQMKEQGAEIDLKVFTEAMQAVYDGKEIKMTEEQAQEVMMKFLQEQQAKAVE 118

Nm_147 GVDIGRSLKQMKEQGAEIDLKVFTEAMQAVYDGKEIKMTEEQAQEVMMKFLQEQQAKAVE 118

Nm_146 GVDIGRSLKQMKEQGAEIDLKVFTEAMQAVYDGKEIKMTEEQAQEVMMKFLQEQQAKAVE 118

Nm_132 GVDIGRSLKQMKEQGAEIDLKVFTEAMQAVYDGKEIKMTEEQAQEVMMKFLQEQQAKAVE 118

Nm_125 GVDIGRSLKQMKEQGAEIDLKVFTEAMQAVYDGKEIKMTEEQAQEVMMKFLQEQQAKAVE 118

Nm_118 GVDIGRSLKQMKEQGAEIDLKVFTEAMQAVYDGKEIKMTEEQAQEVMMKFLQEQQAKAVE 118

Nm_114 GVDIGRSLKQMKEQGAEIDLKVFTEAMQAVYGGKEIKMTEEQAQEVMMKFLQEQQAKAVE 118

Nm_96 GVDIGRSLKQMKEQGAEIDLKVFTEAMQAVYDGKEIKMTEEQAQEVMMKFLQEQQAKAVE 118

Nm_112 GVDIGRSLKQMKEQGAEIDLKVFTEAMQAVYDGKEIKMTEEQAQEVMMKFFQEQQAKAVE 118

Nm_110 GVDIGRSLKQMKEQGAEIDLKVFTEAMQAVYDGKEIKMTEEQAQEVMMKFLQEQQAKAVE 118

Nm_94 GVDIGRSLKQMKEQGAEIDLKVFTEAMQAVYDGKEIKMTEEQAQEVMMKFLQEQQAKAVE 118

Nm_109 GVDIGRSLKQMKEQGAEIDLKVFTEAMQAVYDGKEIKMTEEQAQEVMMKFLQEQQAKAVE 118

Nm_5 GVDIGRSLKQMKEQGAEIDLKVFTEAMQAVYDGKEIKMTEEQAQEVMMKFLQEQQAKAVE 118

Nm_74 GVDIGHSLKQMKEQGAEIDLKVFTEAMQAVYDGKEIKMTEEQAQEVMMKFLQEQQAKAVE 118

Nm_69 GVDIGRSLKQMKEQGAEIDLKVFTEAMQAVYDGKEIKMTEEQAQEVMMKFLQEQQAKAVE 118

Nm_219 GVDIGRSLKQMKEQGAEIDLKVFTEAMQAVYDGKEIKMTEEQAQEVMMKFLQEQQAKAVE 118

Nm_217 GVDIGRSLKQMKEQGAEIDLKVFTEAMQAVYDGKEIKMTEEQAQEVMMKFLQEQQAKAVE 118

Nm_216 GVDIGRSLKQMKEQGAEIDLKVFTEAMQAVYDGKEIKMTEEQAQEVMMKFLQEQQAKAVE 118

Nm_31 GVDIGRSLKQMKEQGAEIDLKVFTEAMQAVYDGKEIKMTEEQAQEVMMKFLQEQQAKAVE 118

Nm_26 GVDIGRSLKQMKEQGAEIDLKVFTEAMQAVYDGKEIKMTEEQAQEVMMKFLQEQQAKAVE 118

Nm_4 GVDIGRSLKQMKEQGAEIDLKVFTEAMQAVYDGKEIKMTEEQAQEVMMKFLQEQQAKAVE 118

Nm_11 GVDIGRSLKQMKEQGAEIDLKVFTEAMQAVYDGKEIKMTEEQAQEVMMKFLQEQQAKAVE 118

*****:***************** :****:* .****:****.*******::**:***:*

Nm_113 KHKADAKANKEKGEAFLKENAAKDGVKTTASGLQYKITKQGEGKQPTKDDIVTVEYEGRL 179

Nm_229 KHKADAKANKEKGEAFLKENAAKEGVKTTASGLQYKITKQGEGKQPTKDDIVTVEYEGRL 178

Nm_27 KHKADAKANKEKGEAFLKENAGKEGVKTTASGLQYKITKQGEGKQPTKDDIVTVEYEGRL 180

Nm_319 KHKADAKANKEKGEAFLKENAGKESVKTTASGLQYKITKQGEGKQPTKDDIVTVEYEGRL 174

Ng_200 KHKADAKANKEKGEAFLKENAAKDGVKTTASGLQYKITKQGEGKQPTKDDIVTVEYEGRL 175

Ng_56 KHKADAKANKEKGEAFLKENAAKDGVKTTASGLQYKITKQGEGKQPTKDDIVTVEYEGRL 178

Ng_208 KHKADAKANKEKGEAFLKENAAKDGVKTTASGLQYKITKQGEGKQPTKDDIVTVEYEGRL 178

Ng_207 KHKADAKANKEKGEAFLKENAAKDGVKTTASGLQYKITKQGEGKQPTKDDIVTVEYEGRL 178

Ng_203 KHKADAKANKEKGEAFLKENAAKDGVKTTASGLQYKITKQGEGKQPTKDDIVTVEYEGRL 178

Ng_204 KHKADAKANKEKGEAFLKENAAKDGVKTTASGLQYKITKQGEGKQPTKDDIVTVEYEGRL 178

Ng_137 KHKADAKANKEKGEAFLKENAAKDGVKTTASGLQYKITKQGEGKQPTKDDIVTVEYEGRL 178

Ng_225 KHKADAKANKEKGEAFLKENAAKDGVKTTASGLQYKITKQGEGKQPTKDDIVTVEYEGRL 178

Ng_8 KHKADAKANKEKGEAFLKENAAKDGVKTTASGLQYKITKQGEGKQPTKDDIVTVEYEGRL 178

Ng_35 KHKADAKANKEKGEAFLKENAAKDGVKTTASGLQYKITKQGEGKQPTKDDIVTVEYEGRL 178

Ng_209 KHKADAKANKEKGEAFLKENAAKDGVKTTASGLQYKITKQGEGKQPTKDDIVTVEYEGRL 178

Ng_140 KHKADAKANKEKGEAFLKENAAKDGVKTTASGLQYKITKQGKGKQPTKDDIVTVEYEGRL 178

Ng_202 KHKADAKANKEKGEAFLKENAAKDGVKTTASGLQYKITKQGKGKQPTKDDIVTVEYEGRL 178

Ng_409 KHKADAKANKEKGEAFLKENAAKDGVKTTASGLQYKITKQGKGKQPTKDDIVTVEYEGRL 178

Ng/Nm_10 KHKADAKANKEKGEAFLKENAAKDGVKTTASGLQYKITKQGKGKQPTKDDIVTVEYEGRL 178

Ng_211 KHKADAKANKEKGEAFLKENAAKDGVKTTASGLQYKITKQGKGKQPTKDDIVTVEYEGRL 178

Ng_205 KHKADAKANKEKGEAFLKENAAKDGVKTTASGLQYKITKQGKGKQPTKDDIVTVEYEGRL 178

Nm_324 KHKADAKANKEKGEAFLKENASKDGVKTTASGLQYKITKQGEGKQPTKDDIVTVEYEGRL 179

Nm_15 KHKADAKANKEKGEAFLKENAAKEGVKTTASGLQYKITKQGEGKQPTKDDIVTVEYEGRL 179

Nm_116 KHKADAKANKEKGEAFLKENAAKDGVKTTASGLQYKITKQGEGKQPTKDDIVTVEYEGRL 178

Nm_295 KHKADAKANKEKGEAFLKENAAKDGVKTTASGLQYKITKQGEGKQPTKDDIVTVEYEGRL 174

Nm_6 KHKADAKANKEKGEAFLKENAGKESVKTTASGLQYKITKQGEGKQPTKDDIVTVEYEGRL 178

Nm_127 KHKADAKANKEKGEAFLKENAGKESVKTTASGLQYKITKQGEGKQPTKDDIVTVEYEGRL 178

Nm_130 KHKAEAKANKEKGEAFLKENAAKDGVKTTASGLQYKITKQGEGKQPTKDDIVTVEYEGRL 174

Nm_107 KHKADAKANKEKGEAFLKENAGKESVKTTASGLQYKITKQGEGKQPTKDDIVTVEYEGRL 174

Nm_419 KHKADAKANKEKGEAFLKENAAKDGVKTTASGLQYKITKQGEGKQPSKDDIVTVEYEGRL 174

Nm_366 KHKADAKANKEKGEAFLKENAAKDGVKTTASGLQYKITKQGEGKQPSKDDIVTVEYEGRL 174

Nm_302 KHKADAKANKEKGEAFLKENAAKDGVKTTASGLQYKITKQGESKQPSKDDIVTVEYEGRL 174

Nm_294 KHKADAKANKEKGEAFLKENAAKDGVKTTASGLQYKITKQGEGKQPTKDDIVTVEYEGRL 174

Nm_287 KHKADAKANKEKGEAFLKENAAKDGVKTTASGLQYKITKQGEGKQPSKDDIVTVEYEGRL 174

Nm_259 KHKADAKANKEKGEAFLKENAAKDGVKTTASGLQYKITKQGEGKQPSKDDIVTVEYEGRL 174

Nm_247 KHKADAKANKEKGEAFLKENAAKDGVKTTASGLQYKITKQGEGKQPTKDDIVTVEYEGRL 174

Nm_240 KHKADAKANKEKGEAFLKENAAKDGVKTTASGLQYKITKQGEGKQPTKDDIVTVEYEGRL 174

Nm_189 KHKADAKANKEKGEAFLKENTAKDGVKTTASGLQYKITKQGEGKQPSKDDIVTVEYEGRL 174

Nm_145 KHKADAKANKEKGEAFLKENAAKDGVKTTASGLQYKITKQGEGKQPTKDDIVTVEYEGRL 174

Nm_106 KHKADAKANKEKGEAFLKENAAKDGVKTTASGLQYKITKQGEGKQPSKDDIVTVEYEGRL 174

Nm-105 KHKADAKANKEKGEAFLKENAAKDGVKTTASGLQYKITKQGEGKQPSKDDIVTVEYEGRL 174

Nm_222 KHKADAKANKEKGEAFLKENAAKDGVKTTASGLQYKITKQSEGKQPTKDDIVTVEYEGRL 174

Nm_215 KHKADAKANKEKGEAFLKENAAKDGVKTTASGLQYKITKQGEGKQPSKDDIVTVEYEGRL 174

Nm_1 KHKADAKANKEKGEAFLKENAAKDGVKTTASGLQYKITKQGEGKQPSKDDIVTVEYEGRL 174

Nm_7 KHKAEAKANKEKGEAFLKENAAKDGVKTTASGLQYKITKQGEGKQPTKDDIVTVEYEGRL 174

Nm_22 KHKADAKANKEKGEAFLKENAAKDGVKTTASGLQYKITKQGEGKQPTKDDIVTVEYEGRL 174

Nm_141 KHKADAKANKEKGEAFLKENAAKDGVKTTASGLQYKITKQGEGKQPSKDDIVTVEYEGRL 178

Nm_374 KHKADAKANKEKGEAFLKENAAKEGVKTTASGLQYKITKQGEGKQPTKDDIVTVEYEGRL 178

Nm_24 KHKADAKANKEKGEAFLKENAAKDGVKTTASGLQYKITKQGEGKQPTKDDIVTVEYEGRL 178

Nm_3 KHKADAKANKEKGEAFLKENAAKDGVKTTASGLQYKITKQGEGKQPTKDDIVTVEYEGRL 178

Ng/Nm_63 KHKADAKANKEKGEAFLKENAAKDGVKTTASGLQYKITKQGEGKQPSKDDIVTVEYEGRL 178

Nm_265 KHKADAKANKEKGEAFLKENAAKDGVKTTASGLQYKITKQGEGKQPTKDDIVTVEYEGRL 178

Nm_486 KHKVDAKANKEKGEAFLKENAAKDGVKTTASGLQYKITKQGEGKQPTKDDIVTVEYEGRL 178

Nm_483 KHKADAKANKEKGEAFLKENAAKDGVKTTASGLQYKITKQGEGKQPTKDDIVTVEYEGRL 178

Nm_466 KHKADAKANKEKGEAFLKENAAKDGVKTTASGLQYKITKQGEGKQPTKDDIVTVEYEGRL 178

Nm_465 KHKAEAKANKEKGEAFLKENAAKDGVKTTASGLQYKITKQGEGKQPTKDDIVTVEYEGRL 178

Nm_406 KHKADAKANKEKGKAFLKENAAKDGVKTTASGLQYKITKQGEGKQPTKDDIVTVEYEGRL 178

Nm_405 KHKADAKANKEKGEAFLKENAAKDGVKTTASGLQYKITKQGEGKQPTKDDIVTVEYEGRL 178

Nm_367 KHKADAKANKEKGEAFLKENVAKDGVKTTASGLQYKITKQGEGKQPTKDDIVTVEYEGRL 178

Nm_365 KHKADAKANKEKGEAFLKENAAKDGVKTTASGLQYKITKQGEGKQPTKDDIVTVEYEGRL 178

Nm_331 KHKADAKANKEKGEAFLKENAGKDGVKTTASGLQYKITKQGEGKQPTKDDIVTVEYEGRL 178

Nm_320 KHKADAKANKEKGEAFLKENAAKDGVKTTASGLQYKITKQGEGKQPTKDDIVTVEYEGRL 178

Nm_301 KHKADAKANKEKGEAFLKENAAKDGVKTTASGLQYKITKQGEGKQPTKDDIVTVEYEGRL 178

Nm_197 KHKADAKANKEKGEAFLKENAAKDGVKTTASGLQYKITKQGEGKQPTKDDIVTVEYEGRL 178

Nm_299 KHKADAKANKEKGEAFLKENAAKDGVKTTASGLQYKITKQGEGKQPTKDDIVTVEYEGRL 178

Nm_290 KYKADAKANKEKGEAFLKENAAKDGVKTTASGLQYKITKQGEGKQPTKDDIVTVEYEGRL 178

Nm_257 KHKADAKANKEKGEAFLKENAAKDGVKTTASGLQYKITKQGEGKQPTKDDIVTVEYEGRL 178

Nm_155 KHKADAKANKEKGEAFLKENAAKDGVKTTASGLQYKITKQGEGKQPTKDDIVTVEYEGRL 178

Nm_152 KHKADAKANKEKGEAFLKENAAKDGVKTTASGLQYKITKQGEGKQPTKDDIVTVEYEGRL 178

Nm_148 KHKADAKANKEKGEAFLKENAAKDGVKTTASGLQYKITKQGEGKQPTKDDIVTVEYEGRL 178

Nm_147 KHKADAKANKEKGEAFLKENAAKDGVKTTASGLQYKITKQGEGKQPTKDDIVTVEYEGRL 178

Nm_146 KHKADAKANKEKGEAFLKENAAKDGVKTTASGLQYKITKQGEGKQPTKDDIVTVEYEGRL 178

Nm_132 KHKADAKANKEKGEAFLKENAAKDGVKTTASGLQYKITKQGEGKQPTKDDIVTVEYEGRL 178

Nm_125 KHKADAKANKEKGEAFLKENAAKDGVKTTASGLQYKITKQGEGKQPTKDDIVTVEYEGRL 178

Nm_118 KHKADAKANKEKGEAFLKENAAKDGVKTTASGLQYKITKQGEGKQPTKDDIVTVEYEGRL 178

Nm_114 KHKADAKANKEKGEAFLKENAAKDGVKTTASGLQYKITKQGEGKQPTKDDIVTVEYEGRL 178

Nm_96 KHKADAKANKEKGEAFLKENAAKDGVKTTASGLQYKITKQGKGKQPTKDDIVTVEYEGRL 178

Nm_112 KHKADAKANKEKGEAFLKENAAKDGVKTTASGLQYKITKQGEGKQPTKDDIVTVEYEGRL 178

Nm_110 KHKADTKANKEKGEAFLKENAAKDGVKTTASGLQYKITKQGEGKQPTKDDIVTVEYEGRL 178

Nm_94 KHKADAKANKEKGEAFLKENAAKDGVKTTASGLQYKITKQGEGKQPTKDDIVTVEYEGRL 178

Nm_109 KHKADAKANKEKGEAFLKENAAKDGVKTTASGLQYKITKQGEGKQPTKDDIVTVEYEGRL 178

Nm_5 KHKADAKANKEKGEAFLKENAAKDGVKTTASGLQYKITKQGEGKQPTKDDIVTVEYEGRL 178

Nm_74 KHKADAKANKEKGEAFLKENAAKDGVKTTASGLQYKITKQGEGKQPTKDDIVTVEYEGRL 178

Nm_69 KHKADAKANKEKGEAFLKENAAKDGVKTTASGLQYKITKQGEGKQPSKDDIVTVEYEGRL 178

Nm_219 KHKADAKANKEKGEAFLKENAAKDGVKTTASGLQYKITKQSEGKQPTKDDIVTVEYEGRL 178

Nm_217 KHKADAKANKEKGEAFLKENAAKDGVKTTASGLQYKITKQGEGKQPTKDDIVTVEYEGRL 178

Nm_216 KHKADAKANKEKGEAFLKENAAKDGVKTTASGLQYKITKQGEGKQPTKDDIVTVEYEGRL 178

Nm_31 KHKADAKANKEKGEAFLKENAAKDGVKTTASGLQYKITKQGEGKQPTKDDIVTVEYEGRL 178

Nm_26 KHKADAKANKEKGEAFLKENAAKDGVKTTASGLQYKITKQGEGKQPTKDDIVTVEYEGRL 178

Nm_4 KHKADAKANKEKGEAFLKENAAKDGVKTTASGLQYKITKQGEGKQPTKDDIVTVEYEGRL 178

Nm_11 KHKADAKANKEKGEAFLKENAAKDGVKTTASGLQYKITKQGEGKQPTKDDIVTVEYEGRL 178

*:*.::*******:******..*:.***************.:.***:*************

Nm_113 IDGTVFDSSKANGGPATFPLSQVIPGWTEGVQLLKEGGEATFYIPSNLAYREQGAGEKIG 239

Nm_229 IDGTVFDSSKANGGPATFPLSQVIPGWTEGVQLLKEGGEATFYIPSNLAYREQGAGEKIG 238

Nm_27 IDGTVFDSSKANGGPATFPLSQVIPGWTEGVQLLKEGGEATFYIPSNLAYREQGAGEKIG 240

Nm_319 IDGTVFDSSKANGGPATFPLSQVIPGWTEGVQLLKEGGEATFYIPSNLAYREQGAGEKIG 234

Ng_200 IDGTVFDSSKANGGPATFPLSQVIPGWTEGVRLLKEGGEATFYIPSNLAYREQGAGEKIG 235

Ng_56 IDGTVFDSSKANGGPATFPLSQVIPGWTEGVRLLKEGGEATFYIPSNLAYREQGAGEKIG 238

Ng_208 IDGTVFDSSKANGGPATFPLSQVIPGWTEGVRLLKEGGEATFYIPSNLAYREQGAGEKIG 238

Ng_207 IDGTVFDSSKANGGPATFPLSQVIPGWTEGVRLLKEGGEATFYIPSNLAYREQGAGEKIG 238

Ng_203 IDGTVFDSSKANGGPATFPLSQVIPGWTEGVRLLKEGGEATFYIPSNLAYREQGAGEKIG 238

Ng_204 IDGTVFDSSKANGGPATFPLNQVIPGWTEGVRLLKEGGEATFYIPSNLAYREQGAGEKIG 238

Ng_137 IDGTVFDSSKANGGPAPFPLSQVIPGWTEGVRLLKEGGEATFYIPSNLAYREQGAGEKIG 238

Ng_225 IDGTVFDSSKANGGPVTFPLSQVIPGWTEGVRLLKEGGEATFYIPSNLAYREQGAGEKIG 238

Ng_8 IDGTVFDSSKANGGPATFPLSQVIPGWTEGVRLLKEGGEATFYIPSNLAYREQGAGEKIG 238

Ng_35 IDGTVFDSSKANGGPATFPLSQVIPGWTEGVRLLKEGGEATFYIPSNLAYREQGAGEKIG 238

Ng_209 IDGTVFDSSKANGGPATFPLSQVIPGWTEGVRLLKEGGEATFYIPSNLAYREQGAGEKIG 238

Ng_140 IDGTVFDSSKANGGPATFPLSQVIPGWTEGVRLLKEGGEATFYIPSNLAYREQGAGEKIG 238

Ng_202 IDGTVFDSSKANGGPATFPLSQVIPGWTEGVRLLKEGGEATFYIPSNLAYREQGAGEKIG 238

Ng_409 IDGIVFDSSKANGGPATFPLSQVIPGWTEGVRLLKEGGEATFYIPSNLAYREQGAGEKIG 238

Ng/Nm_10 IDGTVFDSSKANGGPATFPLSQVIPGWTEGVRLLKEGGEATFYIPSNLAYREQGAGEKIG 238

Ng_211 IDGTVFDSSKANGGPATFPLSQVIPGWTEGVRLLKEGGEATFYIPSNLAYREQGAGEKIG 238

Ng_205 IDGTVFDSSKANGGPATFPLSQVIPGWTEGVRLLKEGGEATFYIPSNLAYREQGAGEKIG 238

Nm_324 IDGTVFDSSKANGGPATFPLSQVIPGWTEGVQLLKEGGEATFYIPSNLAYREQGAGDKIG 239

Nm_15 IDGTVFDSSKANGGPATFPLSQVIPGWTEGVQLLKEGGEATFYIPSNLAYREQGAGEKIG 239

Nm_116 IDGTVFDSSKANGGPVTFPLSQVIPGWTEGVQLLKEGGEATFYIPSNLAYREQGAGDKIG 238

Nm_295 IDGTVFDSSKANGGPVTFPLSQVIPGWTEGVQLLKEGGEATFYIPSNLAYREQGAGDKIG 234

Nm_6 IDGTVFDSSKANGGPVTFPLSQVIPGWTEGVQLLKEGGEATFYIPSNLAYREQGAGDKIG 238

Nm_127 IDGTVFDSSKANGGPVTFPLSQVIPGWTEGVQLLKEGGEATFYIPSNLAYREQGAGDKIG 238

Nm_130 IDGTVFDSSKANGGPATFPLSQVIPGWTEGVQLLKEGGEATFYIPSNLAYREQGAGDKIG 234

Nm_107 IDGTVFDSSKANGGPVTFPLSQVIPGWTEGVQLLKEGGEATFYIPSNLAYREQGAGDKIG 234

Nm_419 IDGTVFDSSKANGGPVTFPLSQVIPGWTEGVQLLKEGGEATFYIPSNLAYREQGAGDKIG 234

Nm_366 IDGTVFDSSKANGGPVTFPLSQVIPGWTEGVQLLKEGGEATFYIPSNLAYREQGAGDKIG 234

Nm_302 IDGTVFDSSKANGGPVTFPLSQVIPGWTEGVQLLKEGGEATFYIPSNLAYREQGAGDKIG 234

Nm_294 IDGTVFDSSKANGGPVTFPLSQMIPGWTEGVQLLKEGGEATFYIPSNLAYREQGAGDKIG 234

Nm_287 IDGTVFDSSKANGGPVTFPLSQVIPGWTEGVQLLKEGGEATFYIPSNLTYREQGAGDKIG 234

Nm_259 IDGTVFDSSKANGGPVTFPLSQVIPGWTEGVQLLKEGGEATFYIPSNLAYREQGAGDKIG 234

Nm_247 IDGTVFDSSKANGGPVTFPLSQVIPGWTEGVQLLKEGGEATFYIPSNLAYREQGAGDKIG 234

Nm_240 IDGTVFDSSKANGGPVTFPLSQVILGWTEGVQLLKEGGEATFYIPSNLAYREQGAGDKIG 234

Nm_189 IDGTVFDSSKANGGPVTFPLSQVIPGWTEGVQLLKEGGEATFYIPSNLAYREQGAGDKIG 234

Nm_145 IDGTVFDSSKANGGTVTFPLSQVIPGWTEGVQLLKEGGEATFYIPSNLAYREQGAGDKIG 234

Nm_106 IDGTVFDSSKANGGPVTFPLSQVIPGWTEGVQLLKEGGEATFYIPSNLAYREQGAGDKIG 234

Nm-105 IDGTVFDSSKANGGPVTFPLSQVIPGWTEGVQLLKEGGEATFYIPSNLAYREQGAGDKIG 234

Nm_222 IDGTVFDSSKANGGPVTFPLSQVIPGWTEGVQLLKEGGEATFYIPSNLAYREQGAGDKIG 234

Nm_215 IDGTVFDSSKANGGPVTFPLSQVIPGWTEGVQFLKEGGEATFYIPSNLAYREQGAGDKIG 234

Nm_1 IDGTVFDSSKANGGPVTFPLSQVIPGWTEGVQLLKEGGEATFYIPSNLAYREQGAGDKIG 234

Nm_7 IDGTVFDSSKANGGPVTFPLSQVIPGWTEGVQLLKEGGEATFYIPSNLAYREQGAGDKIG 234

Nm_22 IDGTVFDSSKANGGPVTFPLSQVIPGWTEGVQLLKEGGEATFYIPSNLAYREQGAGDKIG 234

Nm_141 IDGTVFDSSKANGGTVTFPLSQVIPGWTEGVQLLKEGGEATFYIPSNLAYREQGAGEKIG 238

Nm_374 IDGTVFDSSKANGGPATFPLSQVIPGWTEGVQLLKEGGEATFYIPSNLAYREQGAGDKIG 238

Nm_24 IDGTVFDSSKANGGPVTFPLSQVIPGWTEGVQLLKEGGEATFYIPSNLAYREQGAGDKIG 238

Nm_3 IDGTVFDSSKANGGPVTFPLSQVILGWTEGVQLLKEGGEATFYIPSNLAYREQGAGDKIG 238

Ng/Nm_63 IDGTVFDSSKANGGPVTFPLSQVIPGWTEGVQLLKEGGEATFYIPPNLAYREQGAGDKIG 238

Nm_265 IEGTVFDSSKANGGPVTFPLSQVIPGWTEGVQLLKEGGEATFYIPSNLAYREQGAGDKIG 238

Nm_486 IDGTVFDSSKANGGPVTFPLSQVIPGWTEGVQLLKEGGEATFYIPSNLAYREQGAGDKIG 238

Nm_483 IDGTVFDSSKANGGPVTFPLSQVIPGWTEGVQLLKEGGEATFYIPSNLAYREQGAGDKIG 238

Nm_466 IDDTVFDSSKANGGPVTFPLSQVIPGWTEGVQLLKEGGEATFYIPSNLAYREQGAGDKIG 238

Nm_465 IDGTVFDSSKANGGPVTFPLSQVIPGWTEGVQLLKEGGEATFYIPSNLAYREQGAGDKIG 238

Nm_406 IDGTVFDSSKANGGPVTFPLSQVIPGWTEGVQLLKEGGEATFYIPSNLAYREQGAGDKIG 238

Nm_405 IDGTVFDSSKANGGPVTFPLSQVIPGWTEGVQLLKEGGEATFYIPSNLAYREQGAGDKIG 238

Nm_367 IDGTVFDSSKANGGPVTFPLSQVIPGWTEGVQLLKEGGEATFYIPSNLAYREQGAGDKIG 238

Nm_365 IDGTVFDSSKANGGPVTFPLSQVIPGWTEGVQLLKEGGEATFYIPSNLAYREQGAGDKIG 238

Nm_331 IDGTVFDSSKANGGPVTFPLSQVIPGWTEGVQLLKEGGEATFYIPSNLAYREQGAGDKIG 238

Nm_320 IDGTVFDSSKANGGPVTFPLSQVIPGWTEGVQLLKEGGEATFYIPSNLAYREQGAGDKIG 238

Nm_301 IDGTVFDSSKANGGPVTFPLSQVIPGWTEGVQLLKEGGEATFYIPSNLAYREQGAGDKIG 238

Nm_197 IDGTVFDSSKANGGPVTFPLSQVIPGWTEGVQLLKEGGEATFYIPSNLAYREQGAGDKIG 238

Nm_299 IDGTVFDSGKANGGPVTFPLSQVIPGWTEGVQLLKEGGEATFYIPSNLAYREQGAGDKIG 238

Nm_290 IDGTVFDSSKANGGPVTFPLSQVIPGWTEGVQLLKEGGEATFYIPSNLAYREQGAGDKIG 238

Nm_257 IDGTVFDSSKANGGTVTFPLSQVIPGWTEGVQLLKEGGEATFYIPSNLAYREQGAGDKIG 238

Nm_155 IGGTVFDSSKANGGPVTFPLSQVIPGWTEGVQLLKEGGEATFYIPSNLAYREQGAGDKIG 238

Nm_152 IDGTVFDSSKANGGTVTFPLSQVIPGWTEGVQLLKEGGEATFYIPSNLAYREQGAGDKIG 238

Nm_148 IDGTVFDSSKANGGPVTFPLSQVIPGWTEGVQLLKEGGEATFYIPSNLAYREQGAGDKIG 238

Nm_147 IDGTVFDSSKANGGPVTFPLSQVIPGWTEGVQLLKEGGGATFYIPSNLAYREQGAGDKIG 238

Nm_146 IDGTVFDSSKANGGPVTFPLSQVIPGWTEGVQLLKEGGEATFYIPSNLAYREQGSGDKIG 238

Nm_132 IDGTVFDSSKANGGTVTFPLSQVIPGWTEGVQLLKEGGEATFYIPSNLAYREQGAGDKIG 238

Nm_125 IDGTVFDSSKANGGPVTFPLSQVIPGWTEGVQLLKEGGEATFYIPSNLAYREQGAGDKIG 238

Nm_118 IDGTVFDSSKANGGPVTFPLSQVIPGWTEGVQLLKEGGEATFYIPSNLAYREQGAGDKIG 238

Nm_114 IDGTVFDSSKANGGPVTFPLSQVIPGWTEGVQLLKEGGEATFYIPSNLAYREQGAGDKIG 238

Nm_96 IDGTVFDSSKANGGTVTFPLSQVIPGWTEGVQLLKEGGEATFYIPSNLAYREQGAGDKIG 238

Nm_112 IDGTVFDSSKANGGPVTFPLSQVIPGWTEGVQLLKEGGEATFYIPSNLAYREQGAGDKIG 238

Nm_110 IDGTVFDSSKANGGPVTFPLSQVIPGWTEGVQLLKEGGEATFYIPSNLAYREQGAGDKIG 238

Nm_94 IDGTVFDSSKANGGPVTFPLSQVIPGWTEGVQLLKEGGEATFYIPSNLAYREQGAGDKIG 238

Nm_109 IDGTVFDSSKANGGPVTFPLSQVIPGWTEGVQLLKEGGEATFYIPSNLAYREQGAGDKIG 238

Nm_5 IDGTVFDSSKANGGPVTFPLSQVIPGWTEGVQLLKEGGEATFYIPSNLAYREQGAGDKIG 238

Nm_74 IDGTVFDSSKANGGPVTFPLSQVIPGWTEGVQLLKEGGEATFYIPSNLAYREQGAGDKIG 238

Nm_69 IDGTVFDSSKANGGPVTFPLSQVIPGWTEGVQLLKEGGEATFYIPSNLAYREQGAGDKIG 238

Nm_219 IDGTVFDSSKANGGPVTFPLSQVIPGWTEGVQLLKEGGEATFYIPSNLAYREQGAGDKIG 238

Nm_217 IDGTVFDSSKANGGPVTFPLSQVIPGWTEGVQLLKEGGEATFYIPSNLAYREQGAGDKIG 238

Nm_216 IDGTVFDSSKANGGPVTFPLSQVIPGWTEGVQLLKEGGEATFYIPSNLAYREQGAGEKIG 238

Nm_31 IDGTVFDSSKANGGPVTFPLSQVIPGWTEGVQLLKEGGEATFYIPSNLAYREQGAGDKIG 238

Nm_26 IDGTVFDSSKANGGTVTFPLSQVIPGWTEGVQLLKEGGEATFYIPSNLAYREQGAGDKIG 238

Nm_4 IDGTVFDSSKANGGPVTFPLSQVIPGWTEGVQLLKEGGEATFYIPSNLAYREQGAGDKIG 238

Nm_11 IDGTVFDSSKANGGPVTFPLSQVILGWTEGVQLLKEGGEATFYIPSNLAYREQGAGDKIG 238

* . ****.***** . ***.*:* ******::***** ****** **:*****:*:***

Nm_113 PNSTLVFDVKLVKVGAPGNASAQQPAQVDIKKVN 273

Nm_229 PNSTLVFDVKLVKVGAPENASAQQPAQVDIKKVN 272

Nm_27 PNSTLVFDVKLVKVGAPENASAQQPAQVDIKKVN 274

Nm_319 PNSTLVFDVKLVKVGAPGNASAQQPAQVDIKKVN 268

Ng_200 PNATLVFDVKLVKIGAPENAPAKQPDQVDIKKVN 269

Ng_56 PNATLVFDVKLVKIGAPENAPAKQPDQVDIKKVN 272

Ng_208 PNATLVFDVKLVKIGAPENAPAKQPDQVDIKKVN 272

Ng_207 PNATLVFDVKLVKIGAPENAPAKQPDQVDIKKVN 272

Ng_203 PNATLVFDVKLVKIGAPENAPAKQPDQVDIKKVN 272

Ng_204 PNATLVFDVKLVKIGAPENAPAKQPDQVDIKKVN 272

Ng_137 PNATLVFDVKLVKIGAPENAPAKQPDQVDIKKVN 272

Ng_225 PNATLVFDVKLVKIGAPENAPAKQPDQVDIKKVN 272

Ng_8 PNATLVFDVKLVKIGAPENAPAKQPDQVDIKKVN 272

Ng_35 PNATLVFDVKLVKIGAPENAPAKQPDQVDIKKVN 272

Ng_209 PNATLVFDVKLVKIGAPENAPAKQPDQVDIKKVN 272

Ng_140 PNATLVFDVKLVKIGAPENAPAKQPDQVDIKKVN 272

Ng_202 PNATLVFDVKLVKIGAPENAPAKQPDQVDIKKVN 272

Ng_409 PNATLVFDVKLVKIGAPENAPAKQPDQVDIKKVN 272

Ng/Nm_10 PNATLVFDVKLVKIGAPENAPAKQPDQVDIKKVN 272

Ng_211 PNATLVFDVKLVKIGAPENAPAKQPDQVDIKKVN 272

Ng_205 PNATLVFDVKLVKIGAPENAPAKQPDQVDIKKVN 272

Nm_324 PNSTLVFDVKLVKVGAPENAPAQQPVQVDVKKVN 273

Nm_15 PNATLVFDVKLVKVGAPENASAQQPVQVDVKKVN 273

Nm_116 PNATLVFDVKLVKVGAPENAPTQQPVQVDVKKVN 272

Nm_295 PNATLVFDVKLVKVGAPENAPTQQPVQVDVKKVN 268

Nm_6 PNATLVFDVKLVKIGAPENAPAKQPAQVDIKKVN 272

Nm_127 PNATLVFDVKLVKIGAPENAPAKQPAQVDIKKVN 272

Nm_130 PNATLVFDVKLVKVGAPENAPAKQPVQVDIKKVN 268

Nm_107 PNATLVFDVKLVKIGAPENAPAKQPAQVDIKKVN 268

Nm_419 PNATLVFDVKLVKIGAPENAPAKQPAQVDIKKVN 268

Nm_366 PNATLVFDVKLVKIGAPENVPAKQPAQVDIKKVN 268

Nm_302 PNATLVFDVKLVKIGAPENAPAKQPAQVDIKKVN 268

Nm_294 PNATLVFDVKLVKIGAPENAPAKQPAQVDIKKVN 268

Nm_287 PNATLVFDVKLVKIGAPENAPAKQPAQVDIKKVN 268

Nm_259 PNATLVFDVKLVKIGAPENAPAKQPAQVDIKKVN 268

Nm_247 PNATLVFDVKLVKIGAPENAPAKQPAQVDIKKVN 268

Nm_240 PNATLVFDVKLVKIGAPENAPAKQPAQVDIKKVN 268

Nm_189 PNATLVFDVKLVKIGAPENAPAKQPAQVDIKKVN 268

Nm_145 PNATLVFDVKLVKIGAPENAPAKQPAQVDIKKVN 268

Nm_106 PNATLVFDVKLVKIGSPENAPAKQPAQVDIKKVN 268

Nm-105 PNATLVFDVKLVKIGAPENAPAKQPAQVDIKKVN 268

Nm_222 PNATLVFDVKLVKIGAPENAPAKQPAQVDIKKVN 268

Nm_215 PNATLVFDVKLVKIGAPENAPAKQPAQVDIKKVN 268

Nm_1 PNATLVFDVKLVKIGAPENAPAKQPAQVDIKKVN 268

Nm_7 PNATLVFDVKLVKIGAPENAPAKQPAQVDIKKVN 268

Nm_22 PNATLVFDVKLVKIGAPENAPAKQPAQVDIKKVN 268

Nm_141 PNATLVFDVKLVKVGAPENAPAQQPVQVDVKKVN 272

Nm_374 PNATLVFDAKLVKVGAPENAPAKQPVQVDIKKVN 272

Nm_24 PNATLVFDVKLVKIGAPENAPAKQPAQVDIKKVN 272

Nm_3 PNATLVFDVKLVKIGAPENAPAKQPAQVDIKKVN 272

Ng/Nm_63 PNATLVFDVKLVKIGAPENAPAKQPAQVDIKKVN 272

Nm_265 PNATLVFDVKLVKIGAPENAPAKQPAQVDIKKVN 272

Nm_486 PNATLVFDVKLVKIGAPENAPAKQPAQVDIKKVN 272

Nm_483 PNATLVFDVKLVKIGAPENAPAKQPAQVDIKKVN 272

Nm_466 PNATLVFDVKLVKIGAPENAPAKQPAQVDIKKVN 272

Nm_465 PNATLVFDVKLVKIGAPENAPAKQPAQVDIKKVN 272

Nm_406 PNATLVFDVKLVKIGAPENAPAKQPAQVDIKKVN 272

Nm_405 PNATLVFDVKLVKIGAPENAPAKQPAQVDIKKVN 272

Nm_367 PNATLVFDVKLVKIGAPENAPAKQPAQVDIKKVN 272

Nm_365 PNATLVFDVKLVKIGAPENAPAKQPAQVDIKKVN 272

Nm_331 PNATLVFDVKLVKIGAPENAPAKQPAQVDIKKVN 272

Nm_320 PNATLVFDVKLVKIGAPENAPAKQPAQVDIKKVN 272

Nm_301 PNATLVFDVKLVKIGAPENASAKQPAQVDIKKVN 272

Nm_197 PNATLVFDVKLVKIGAPENAPAKQPAQVDIKKVN 272

Nm_299 PNATLVFDVKLVKIGAPENAPAKQPAQVDIKKVN 272

Nm_290 PNATLVFDVKLVKIGAPENAPAKQPAQVDIKKVN 272

Nm_257 PNATLVFDVKLVKIGAPENAPSKQPAQVDIKKVN 272

Nm_155 PNATLVFDVKLVKIGAPENAPAKQPAQVDIKKVN 272

Nm_152 PNATLVFDVKLVKIGAPENAPAKQPAQVDIKKVN 272

Nm_148 PNATLVFDVKLVKIGAPENAPAKQPVQVDIKKVN 272

Nm_147 PNATLVFDVKLVKIGAPENAPAKQPAQVDIKKVN 272

Nm_146 PNATLVFDVKLVKIGAPENAPAKQPAQVDIKKVN 272

Nm_132 PNATLVFDVKLVKIGAPENAPAKQPAQVDIKKVN 272

Nm_125 PNTTLVFDVKLVKIGAPENAPAKQPAQVDIKKVN 272

Nm_118 PNATLVFDVKLVKIGAPEKAPAKQPAQVDIKKVN 272

Nm_114 PNATLVFDVKLVKIGAPENAPAKQPAQVDIKKVN 272

Nm_96 PNATLVFDVKLVKIGAPENAPAKQPAQVDIKKVN 272

Nm_112 PNATLVFDVKLVKIGAPENAPAKQPAQVDIKKVN 272

Nm_110 PNATLVFDVKLVKIGAPENAPAKQPAQVDIKKVN 272

Nm_94 PNATLVFDVKLVKIGAPENAPAKQPAQVDIKKVN 272

Nm_109 PNATLVFDVKLVKIGAPENAPAKQPAQVDIKKVN 272

Nm_5 PNATLVFDVKLVKIGAPENAPAKQPAQVDIKKVN 272

Nm_74 PNATLVFDVKLVKIGAPENAPAKQPAQVDIKKVN 272

Nm_69 PNATLVFDVKLVKIGAPENAPAKQPAQVDIKKVN 272

Nm_219 PNATLVFDVKLVKIGAPENAPAKQPAQVDIKKVN 272

Nm_217 PNATLVFDVKLVKIGAPENAPAKQSAQVDIKKVN 272

Nm_216 PNATLVFDVKLVKIGAPENAPAKQPAQVDIKKVN 272

Nm_31 PNATLVFDVKLVKIGAPENAPAKQPAQVDIKKVN 272

Nm_26 PNATLVFDVKLVKIGAPENAPAKQPAQVDIKKVN 272

Nm_4 PNATLVFDVKLVKIGAPENAPAKQPAQVDIKKVN 272

Nm_11 PNATLVFDVKLVKIGAPENAPAKQPAQVDIKKVN 272

**:*****.****:*:* :. ::* ***:****

**Supplementary Fig 2. Clustal alignment of the non-redundant translated amino acid sequences for MIP proteins corresponding to known alleles found in meningococcal and gonococcal isolates in the pubmlst.org/*Neisseria* database.** PubMLST database (<http://pubmlstorg/perl/bigsdb/bigsdbpl?db=pubmlst_neisseria_isolates>) was accessed January 2018. Amino acid sequence alignments were generated using Clustal Omega (<http://www.ebi.ac.uk/Tools/msa/clustalo/>). Nm, meningococcus; Ng, gonococcus. ***** (asterisk) denotes fully conserved amino acid residue; **:** (colon) indicates conservation between groups of strongly similar properties; **.** (period) denotes conservation between groups of weakly similar properties.
