# Supplementary material for: Immunization with recombinant truncated Neisseria meningitidis-Macrophage Infectivity Potentiator (rT-Nm-MIP) protein induces murine antibodies that are cross-reactive and bactericidal for Neisseria gonorrhoeae
Source: Vaccine. 2018 Jun 22;36(27):3926–36. doi: 10.1016/j.vaccine.2018.05.069 (PMC6018565; doi:10.1016/j.vaccine.2018.05.069)
Supplement: Supplementary Fig. 4 — Comparison of M2 Nm-MIP (Type I) to M1, M6, M7 Nm-MIP (Types II-IV) and M8, M10 and M35 Ng-MIP proteins. Percentage of identity of M1, M6, M7 Nm-MIP proteins (Types II-IV) and M8, M10 and M35 Ng-MIP proteins to M2 Nm-MIP (Type I) N-terminal domain (amino acid residues 1-142) and C-terminal domain (amino acid residues 143-272) are specified. M2 Nm-MIP leader sequence (amino acid residues 1-22) is coloured in black and the FKBP domain is striped (amino acid residues 166-252). [file mmc3.pptx]

## Slide 1
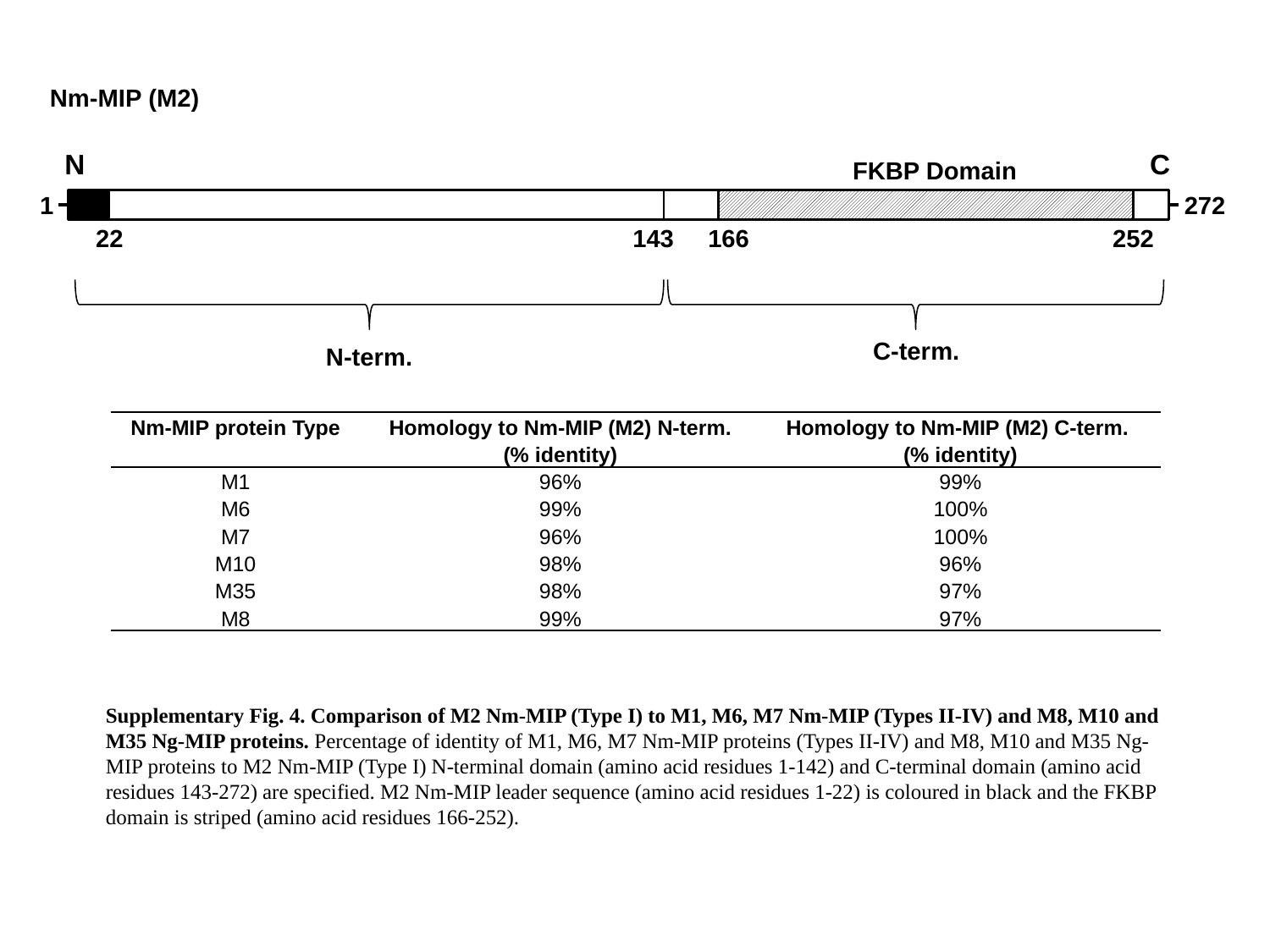

Nm-MIP (M2)
N
C
FKBP Domain
1
272
22
143
166
252
C-term.
N-term.
| Nm-MIP protein Type | Homology to Nm-MIP (M2) N-term. (% identity) | Homology to Nm-MIP (M2) C-term. (% identity) |
| --- | --- | --- |
| M1 | 96% | 99% |
| M6 | 99% | 100% |
| M7 | 96% | 100% |
| M10 | 98% | 96% |
| M35 | 98% | 97% |
| M8 | 99% | 97% |
Supplementary Fig. 4. Comparison of M2 Nm-MIP (Type I) to M1, M6, M7 Nm-MIP (Types II-IV) and M8, M10 and M35 Ng-MIP proteins. Percentage of identity of M1, M6, M7 Nm-MIP proteins (Types II-IV) and M8, M10 and M35 Ng-MIP proteins to M2 Nm-MIP (Type I) N-terminal domain (amino acid residues 1-142) and C-terminal domain (amino acid residues 143-272) are specified. M2 Nm-MIP leader sequence (amino acid residues 1-22) is coloured in black and the FKBP domain is striped (amino acid residues 166-252).
