# Supplementary material for: Immunization with recombinant truncated Neisseria meningitidis-Macrophage Infectivity Potentiator (rT-Nm-MIP) protein induces murine antibodies that are cross-reactive and bactericidal for Neisseria gonorrhoeae
Source: Vaccine. 2018 Jun 22;36(27):3926–36. doi: 10.1016/j.vaccine.2018.05.069 (PMC6018565; doi:10.1016/j.vaccine.2018.05.069)
Supplement: Supplementary Fig. 5 — Analysis of MC58 wild-type (WT), MC58mip and MC58mip::t-nm-mip OM preparations. (A) OM preparations (20 µg) of MC58 WT, mip knock-out and complemented with rT-Nm-MIP strains were reacted with polyclonal rabbit sera (1/400 dilution) raised against full length M2 rNm-MIP Type I [17] in western immunoblotting. Full length WT and truncated Nm-MIP proteins were recognised as a single band of Mr ∼ 29 kDa and ∼14 kDa, respectively (identified by the arrow). MIP was not detected in the MC58mip OM preparation. (B) SDS-PAGE of 20 µg of each OM preparation. [file mmc4.pptx]

## Slide 1
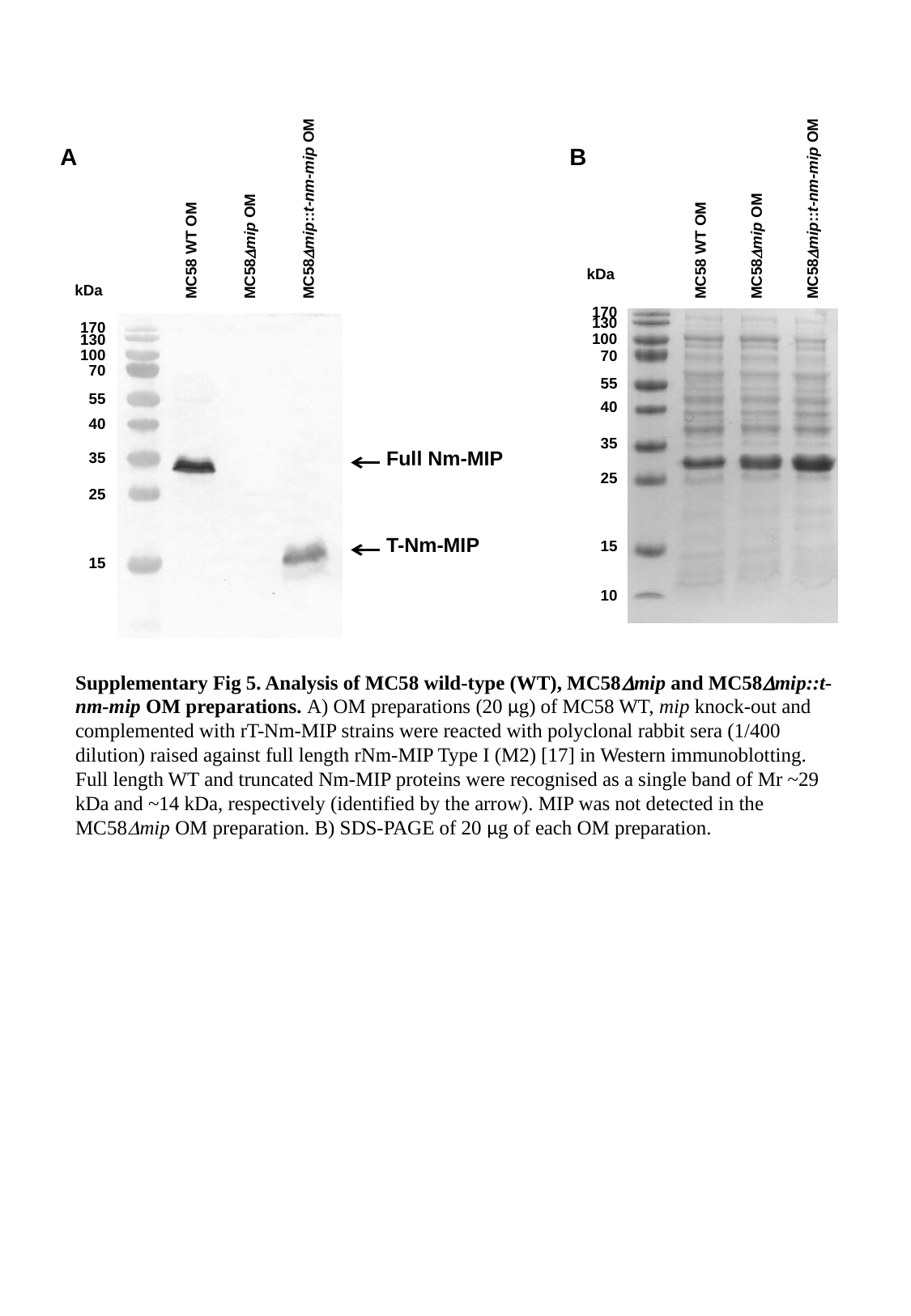

A
B
MC58Δmip::t-nm-mip OM
MC58Δmip::t-nm-mip OM
MC58Δmip OM
MC58Δmip OM
MC58 WT OM
MC58 WT OM
kDa
kDa
170
130
170
100
130
100
70
70
55
55
40
40
35
Full Nm-MIP
35
25
25
T-Nm-MIP
15
15
10
Supplementary Fig 5. Analysis of MC58 wild-type (WT), MC58Δmip and MC58Δmip::t-nm-mip OM preparations. A) OM preparations (20 µg) of MC58 WT, mip knock-out and complemented with rT-Nm-MIP strains were reacted with polyclonal rabbit sera (1/400 dilution) raised against full length rNm-MIP Type I (M2) [17] in Western immunoblotting. Full length WT and truncated Nm-MIP proteins were recognised as a single band of Mr ~29 kDa and ~14 kDa, respectively (identified by the arrow). MIP was not detected in the MC58Δmip OM preparation. B) SDS-PAGE of 20 µg of each OM preparation.
