# Supplementary material for: Immunization with recombinant truncated Neisseria meningitidis-Macrophage Infectivity Potentiator (rT-Nm-MIP) protein induces murine antibodies that are cross-reactive and bactericidal for Neisseria gonorrhoeae
Source: Vaccine. 2018 Jun 22;36(27):3926–36. doi: 10.1016/j.vaccine.2018.05.069 (PMC6018565; doi:10.1016/j.vaccine.2018.05.069)
Supplement: Supplementary Fig. 6 — Western immunobloting of murine antisera to engineered meningococci native and Na-DOC OM. Groups of five BALB/c mice were immunized with three doses of MC58 wild-type (WT) native OM, MC58Δmip OM and complemented MC58Δmip::t-nm-mip OM and the corresponding Na-DOC OM preparations (20 µg/mouse) on days 0, 14, and 28. Groups of five mice were also sham immunized. Pooled murine antisera (1/100 dilution; n = 5 animals) raised against the native and Na-DOC OM preparations were reacted against purified recombinant full length M2 Nm-MIP protein in western blot. rNm-MIP protein was recognised as a single, strong band of Mr ∼ 33 kDa with anti-MC58 WT native OM sera. Lower reactivity was observed with antisera to MC58 Na-DOC OM, complemented MC58 OM and Na-DOC OM. No significant reactivity was observed with antisera raised against MC58mip OM preparation. Sham immunisation sera were non-reactive. [file mmc5.pptx]

## Slide 1
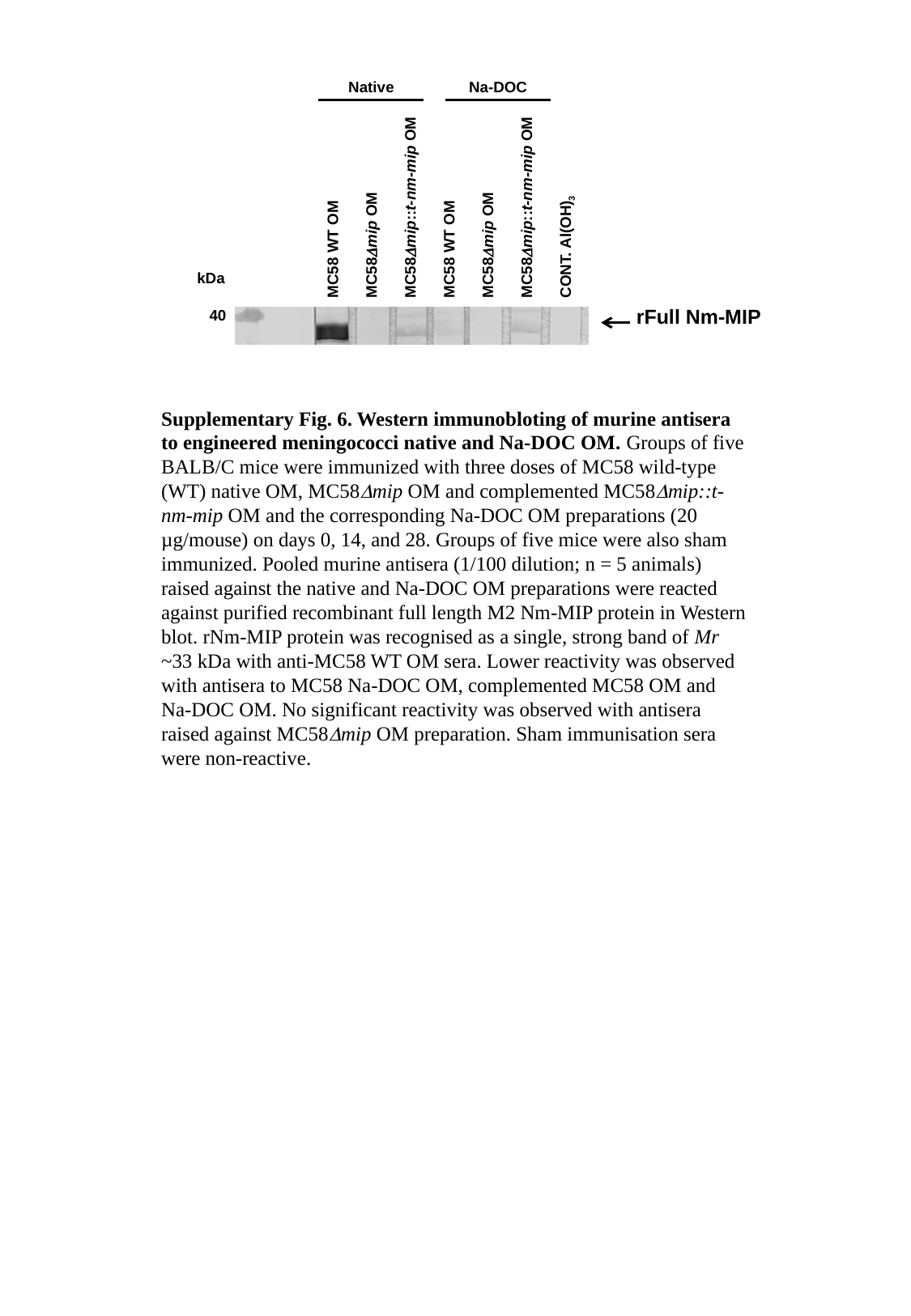

Native
Na-DOC
MC58Δmip::t-nm-mip OM
MC58Δmip::t-nm-mip OM
MC58Δmip OM
MC58Δmip OM
MC58 WT OM
MC58 WT OM
CONT. Al(OH)3
kDa
rFull Nm-MIP
40
Supplementary Fig. 6. Western immunobloting of murine antisera to engineered meningococci native and Na-DOC OM. Groups of five BALB/C mice were immunized with three doses of MC58 wild-type (WT) native OM, MC58Δmip OM and complemented MC58Δmip::t-nm-mip OM and the corresponding Na-DOC OM preparations (20 µg/mouse) on days 0, 14, and 28. Groups of five mice were also sham immunized. Pooled murine antisera (1/100 dilution; n = 5 animals) raised against the native and Na-DOC OM preparations were reacted against purified recombinant full length M2 Nm-MIP protein in Western blot. rNm-MIP protein was recognised as a single, strong band of Mr ~33 kDa with anti-MC58 WT OM sera. Lower reactivity was observed with antisera to MC58 Na-DOC OM, complemented MC58 OM and Na-DOC OM. No significant reactivity was observed with antisera raised against MC58Δmip OM preparation. Sham immunisation sera were non-reactive.
