# Supplementary material for: Immunization with recombinant truncated Neisseria meningitidis-Macrophage Infectivity Potentiator (rT-Nm-MIP) protein induces murine antibodies that are cross-reactive and bactericidal for Neisseria gonorrhoeae
Source: Vaccine. 2018 Jun 22;36(27):3926–36. doi: 10.1016/j.vaccine.2018.05.069 (PMC6018565; doi:10.1016/j.vaccine.2018.05.069)
Supplement: Supplementary tables [file mmc6.docx]

| **Primer Name** | **Sequence (5’ - 3’)** | **Purpose** |
| --- | --- | --- |
|  |  |  |
| F1Fw | CGCGTTTTGGAAATCGTCGG | Construction of *Δmip* (F1) |
| F1Rv | TATTATAAGCTTGATGGATCTTCGCTGTCG | Construction of *Δmip* (F1) |
| F2Fw | TATTATAAGCTTGTCCGAATCCATGCCCG | Construction of *Δmip* (F2) |
| F2Rv | TCAGCCTGCCTTCGTAACGG | Construction of *Δmip* (F2) |
| ErmFw | AATAATAAGCTTTTCAGACGGCCACTAGACTTATTTACTTCG | Construction of *Δmip* (Erm) |
| ErmRv | CGCGATAAGCTTTTACTTATTAAATAATTTATAGC | Construction of *Δmip* (Erm) |
| 1567-NdeI-sens-woHis | GGAATTCCATATGGGCAAAAAAGAAGCCGCCC | Cloning of *rt-nm-mip* |
| 1567-trunc-real-his-Cterm | CCGCTCGAGTCATCAGTGGTGGTGATGATGATGGCCGCCGTC  TTTGGCGGCATTTTCTTTC | Cloning of *rt-nm-mip* |

**Supplementary Table 1.** **List of primers used in this study.**

|  | **Meningococal Serogroup** | | | | | | | | | | | | |  |
| --- | --- | --- | --- | --- | --- | --- | --- | --- | --- | --- | --- | --- | --- | --- |
| **Nm-MIP Allele** | **A** | **B** | **C** | **E** | **H** | **W** | **X** | **Y** | **Z** | **NG** | **ND** | **Totals** | **% of total** | **MIP Protein Type** |
| **2** (+4+12+14+25+62+73+93 +97+98+99+104+123+124 +129+150+153+171+194 +224+237+239+241+242 +243+245+258+261+268 +286+300+311+314+352 +364+377+449+464+485) | 58 | 2909 | 220 | 24 |  | 34 | 33 | 102 | 4 | 222 | 669 | 4275 | 41 | M2 |
| **1** (+108+154+161+172+199 +223+267+284+303+375+450 +458+459+463+469) | 1 | 251 | 995 | 4 |  | 1616 | 2 | 6 | 1 | 96 | 379 | 3351 | 32 | M1 |
| **7** (+68+75+169+220+285) | 146 | 29 | 8 | 1 |  | 27 | 1 | 818 |  | 8 | 95 | 1133 | 11 | M7 |
| **13** (+26+76+151 +277+298+373) | 3 | 335 | 13 |  |  | 5 |  | 174 |  | 10 | 33 | 573 | 5 | M13 |
| **31** (+32+65+67+70+111+121 +221+305+407) | 2 | 97 | 182 | 1 |  | 5 | 2 | 40 | 6 | 9 | 46 | 390 | 4 | M31 |
| **6** (+23+30+128+218) |  | 35 | 23 | 1 |  | 2 |  | 4 |  | 24 | 101 | 190 | 2 | M6 |
| **22** (+57+117+126+149+238 +248+368) |  | 22 | 24 |  | 1 | 30 |  | 75 | 1 | 24 | 12 | 189 | 2 | M22 |
| **63** (+404, 444) |  | 1 |  |  |  | 1 |  | 2 |  | 30 | 81 | 115 | 1 | M63 |
| **11** (+122) | 21 | 1 |  |  |  |  |  |  |  |  | 1 | 23 | 0 |  |
| **66** (+69+475) |  | 8 |  | 1 |  |  |  |  |  | 7 | 6 | 22 | 0 |  |
| **109** |  | 17 |  |  |  |  |  |  |  |  | 1 | 18 | 0 |  |
| **127** |  |  |  |  |  |  |  | 1 |  | 5 | 10 | 16 | 0 |  |
| **116** |  | 12 |  |  |  |  |  |  |  |  | 1 | 13 | 0 |  |
| **5** (+115) |  | 2 | 1 |  |  |  |  |  |  | 1 | 7 | 11 | 0 |  |
| **107** |  | 5 | 1 | 1 |  |  |  |  |  |  | 3 | 10 | 0 |  |
| **216** |  |  |  |  |  |  |  |  |  | 6 |  | 6 | 0 |  |
| **145** (+244) |  | 5 |  |  |  |  |  |  |  |  |  | 5 | 0 |  |
| **105** |  | 1 | 2 |  |  |  |  |  |  |  | 2 | 5 | 0 |  |
| **74** (+95) |  | 4 |  |  |  |  |  |  |  |  |  | 4 | 0 |  |
| **112** (+246) |  | 4 |  |  |  |  |  |  |  |  |  | 4 | 0 |  |
| **94** |  | 4 |  |  |  |  |  |  |  |  |  | 4 | 0 |  |
| **125** |  |  |  |  |  |  |  |  |  |  | 4 | 4 | 0 |  |
| **141** |  |  | 3 |  |  |  |  |  |  |  | 1 | 4 | 0 |  |
| **215** |  |  |  | 1 |  |  |  |  |  | 3 |  | 4 | 0 |  |
| **366** |  |  | 4 |  |  |  |  |  |  |  |  | 4 | 0 |  |
| **302** |  |  |  |  |  |  |  |  |  |  | 3 | 3 | 0 |  |
| **320** |  | 2 |  |  |  |  |  |  |  |  | 1 | 3 | 0 |  |
| **10** |  |  |  |  |  |  |  |  |  |  | 2 | 2 | 0 | M10 |
| **27** |  |  |  |  |  |  |  |  |  |  | 2 | 2 | 0 |  |
| **110** |  | 2 |  |  |  |  |  |  |  |  |  | 2 | 0 |  |
| **197** |  | 2 |  |  |  |  |  |  |  |  |  | 2 | 0 |  |
| **217** |  |  |  |  |  |  |  |  | 2 |  |  | 2 | 0 |  |
| **229** |  | 1 |  |  |  |  |  |  |  | 1 |  | 2 | 0 |  |
| **287** |  |  | 2 |  |  |  |  |  |  |  |  | 2 | 0 |  |
| **3** | 1 |  |  |  |  |  |  |  |  |  |  | 1 | 0 |  |
| **15** |  | 1 |  |  |  |  |  |  |  |  |  | 1 | 0 |  |
| **24** |  | 1 |  |  |  |  |  |  |  |  |  | 1 | 0 |  |
| **96** |  | 1 |  |  |  |  |  |  |  |  |  | 1 | 0 |  |
| **106** |  |  |  |  |  | 1 |  |  |  |  |  | 1 | 0 |  |
| **113** |  | 1 |  |  |  |  |  |  |  |  |  | 1 | 0 |  |
| **114** |  | 1 |  |  |  |  |  |  |  |  |  | 1 | 0 |  |
| **118** |  |  | 1 |  |  |  |  |  |  |  |  | 1 | 0 |  |
| **130** |  |  |  |  |  |  |  |  |  |  | 1 | 1 | 0 |  |
| **132** |  | 1 |  |  |  |  |  |  |  |  |  | 1 | 0 |  |
| **146** |  | 1 |  |  |  |  |  |  |  |  |  | 1 | 0 |  |
| **147** |  | 1 |  |  |  |  |  |  |  |  |  | 1 | 0 |  |
| **148** |  | 1 |  |  |  |  |  |  |  |  |  | 1 | 0 |  |
| **152** |  | 1 |  |  |  |  |  |  |  |  |  | 1 | 0 |  |
| **155** |  | 1 |  |  |  |  |  |  |  |  |  | 1 | 0 |  |
| **189** |  |  |  |  |  | 1 |  |  |  |  |  | 1 | 0 |  |
| **219** |  |  |  |  |  |  |  |  |  | 1 |  | 1 | 0 |  |
| **222** |  |  |  |  |  |  |  |  |  | 1 |  | 1 | 0 |  |
| **240** |  |  |  |  |  | 1 |  |  |  |  |  | 1 | 0 |  |
| **247** |  | 1 |  |  |  |  |  |  |  |  |  | 1 | 0 |  |
| **257** |  | 1 |  |  |  |  |  |  |  |  |  | 1 | 0 |  |
| **259** |  |  |  |  |  | 1 |  |  |  |  |  | 1 | 0 |  |
| **265** |  |  | 1 |  |  |  |  |  |  |  |  | 1 | 0 |  |
| **290** |  | 1 |  |  |  |  |  |  |  |  |  | 1 | 0 |  |
| **294** |  |  |  |  |  |  |  | 1 |  |  |  | 1 | 0 |  |
| **295** |  |  | 1 |  |  |  |  |  |  |  |  | 1 | 0 |  |
| **299** |  | 1 |  |  |  |  |  |  |  |  |  | 1 | 0 |  |
| **301** |  |  |  |  |  |  |  | 1 |  |  |  | 1 | 0 |  |
| **319** |  | 1 |  |  |  |  |  |  |  |  |  | 1 | 0 |  |
| **324** |  | 1 |  |  |  |  |  |  |  |  |  | 1 | 0 |  |
| **331** |  |  | 1 |  |  |  |  |  |  |  |  | 1 | 0 |  |
| **365** |  | 1 |  |  |  |  |  |  |  |  |  | 1 | 0 |  |
| **367** |  | 1 |  |  |  |  |  |  |  |  |  | 1 | 0 |  |
| **374** |  | 1 |  |  |  |  |  |  |  |  |  | 1 | 0 |  |
| **405** |  |  |  |  |  |  |  |  |  |  | 1 | 1 | 0 |  |
| **406** |  |  |  |  |  |  |  |  |  |  | 1 | 1 | 0 |  |
| **419** |  |  |  |  |  | 1 |  |  |  |  |  | 1 | 0 |  |
| **465** |  |  |  |  |  |  |  | 1 |  |  |  | 1 | 0 |  |
| **466** |  | 1 |  |  |  |  |  |  |  |  |  | 1 | 0 |  |
| **483** |  | 1 |  |  |  |  |  |  |  |  |  | 1 | 0 |  |
| **486** |  |  |  |  |  |  |  |  |  | 1 |  | 1 | 0 |  |
| **Totals** | **232** | **3771** | **1482** | **34** | **1** | **1725** | **38** | **1225** | **14** | **449** | **1463** | **10434** | **100** |  |

| **Ng-MIP Allele** | **Total isolates** | **% Total** | **MIP Protein Type** |
| --- | --- | --- | --- |
| **10** (+138+139+256+297+411) | 2274 | 59 | M10 |
| **35** (+210+408+410+412) | 1029 | 27 | M35 |
| **140** (+206) | 180 | 5 | M140 |
| **8** (+34+201) | 177 | 5 | M8 |
| **205** | 118 | 3 | M205 |
| **202** (+212) | 51 | 1 | M202 |
| **203** (+214) | 18 | 0 |  |
| **209** (+213) | 6 | 0 |  |
| **63** | 5 | 0 | M63 |
| **204** | 5 | 0 |  |
| **208** | 5 | 0 |  |
| **200** | 2 | 0 |  |
| **56** | 1 | 0 |  |
| **137** | 1 | 0 |  |
| 207 | 1 | 0 |  |
| 211 | 1 | 0 |  |
| 225 | 1 | 0 |  |
| 409 | 1 | 0 |  |
|  | **3876** | **100** |  |

**Supplementary Table 2. Analysis of NMB1567/NEIS1487 (MIP) alleles for all meningococcal and gonococcal isolates in the pubmlst/*Neisseria*.org database.** Data were collated from <https://pubmlst.org/bigsdb?db=pubmlst_neisseria_isolates> database and examined only isolates for which alleles were identified. Numbers in parenthesis indicate that the alleles produce proteins with identical amino acid sequences. Database was accessed January, 2018. The MIP Type designation scheme was originally based on the isolate frequency for each non-redundant protein, referred to as Types I-IV, with Type I the most abundant Type protein within all *N. meningitidis* isolates reported [13, 17] . We now re-assign the MIP Type designation by referring to the representative allele encoding for a particular non-redundant MIP protein, thus becoming independent of the isolate frequency ranking for each particular MIP type, which varies as the database grows in number of isolates reported. A MIP Type has only been assigned to proteins represented by at least 1% of total isolates. MIP Types M10 and M63 (highlighted in red) are the only two protein types shared by both *N. meningitidis* and *N. gonorrhoeae*. ND, not determined; NG, no serogroup identified.
